# UBE2S as a novel ubiquitinated regulator of p16 and β-catenin to promote bone metastasis of prostate cancer

**DOI:** 10.7150/ijbs.72629

**Published:** 2022-05-16

**Authors:** Shengmeng Peng, Xu Chen, Chaoyun Huang, Chenwei Yang, Minyi Situ, Qianghua Zhou, Yihong Ling, Hao Huang, Ming Huang, Yangjie Zhang, Liang Cheng, Qiang Zhang, Zhenghui Guo, Yiming Lai, Jian Huang

**Affiliations:** 1 Department of Urology, Sun Yat-sen Memorial Hospital, Sun Yat-sen University, Guangzhou 510120, P. R. China.; 2 Guangdong Provincial Key Laboratory of Malignant Tumor Epigenetics and Gene Regulation, Sun Yat-Sen Memorial Hospital, Sun Yat-Sen University, Guangzhou 510120, P. R. China.; 3 Department of Pathology, Sun Yat-sen University Cancer Center, Guangzhou, 510060, P. R. China.; 4 Department of Animal Center, Sun Yat-sen University Cancer Center, Guangzhou, 510060, P. R. China.; 5 Guangdong Provincial Clinical Research Center for Urological Diseases, Guangzhou, Guangdong, 510120, P. R. China.

**Keywords:** UBE2S, Bone metastasis of PCa, p16, β-catenin, K11-linked ubiquitination

## Abstract

Bone metastasis is the main site of metastasis and causes the most deaths in patients with prostate cancer (PCa). The mechanism of bone metastasis is complex and not fully clarified. By RNA sequencing and analysing key pathways in bone metastases of PCa, we found that one of the most important characteristics during PCa bone metastasis was G1/S transition acceleration caused by low protein levels of p16INK4a (p16). Interestingly, we demonstrated that UBE2S bound and degraded p16 through K11- rather than K48- or K63-linked ubiquitination, which accelerated PCa tumour cell G1/S transition *in vivo* and* in vitro*. Moreover, UBE2S also stabilized β-catenin through K11-linked ubiquitination, leading to enhanced migration and invasion of tumour cells in PCa bone metastasis. Based on our cohorts and public databases, UBE2S was overexpressed in bone metastases and positively correlated with a high Gleason score, advanced nodal metastasis status and poor prognosis in PCa. Finally, targeting UBE2S with cephalomannine inhibited proliferation and invasion *in vitro*, and bone metastasis of PCa* in vivo*. This study innovatively discovered that UBE2S plays an oncogenic role in bone metastasis of PCa by degrading p16 and stabilizing β-catenin via K11-linked ubiquitination, suggesting that it may serve as a multipotent target for metastatic PCa treatment.

## Introduction

Prostate cancer (PCa) is the most common tumour in males worldwide 1. The main cause of death of PCa is the occurrence of distant metastasis, of which bone metastasis accounts for approximately 65%-80%. The 1-year survival for PCa with bone metastases decreased from 87% to 47%, while the 5-year survival decreased from 56% to 3% 2. Bone metastasis is a multistage process, including tumour cells escaping from the primary site into the circulation, surviving in the circulation, entering the premetastatic niche, homing to the bone marrow and finally proliferating 3. The molecular mechanism involved in bone metastasis has been partially recognized 4. In PCa, the RANK/RANKL 5 and CXCR4/CXCL12 6 axes have been reported to play a crucial role in bone metastasis by regulating bone remodelling and tumour cell recruitment, respectively. However, the current targeted drugs based on those molecules, such as the RANKL inhibitor denosumab, did not improve the survival of patients with PCa bone metastasis 7. Therefore, it is urgent to further explore the mechanism underlying bone metastasis of PCa and discover potential therapeutic targets. It has been widely reported that cell cycle progression is involved in tumour metastasis. In pancreatic cancer, cell cycle progression is the most important hallmark of metastases 8,9. In PCa, the proteomes of patients with bone metastasis also showed that the BM2 subtype, which defined as high MCM3 scores and low PSA scores, has increased expression of proliferation proteins 10. However, the specific mechanism that mediates tumour cell proliferation in bone metastasis of PCa remains unclear.

The p16 protein, which is encoded by CDKN2A, was found to play an important role as a tumour suppressor in many cancers, such as pancreatic and lung cancer 11. In pancreatic adenocarcinoma, loss of p16 promoted tumor progression and was associated with poor survival 12. In non-small cell lung cancer, p16 inactivation promoted cell proliferation and mediated RB pathway perturbation 13,14. Mechanistically, the p16 protein, as a negative regulator of the cell cycle, inhibits the activity of cyclin D1-dependent kinase 4 or 6 complex, which finally leads to G1/S transition arrest and tumour suppression. Previous studies found that the causes of CDKN2A inactivation in various cancers were mainly point mutation, homozygous deletion or promoter hyper-methylation. Recently, posttranslational modification of p16 has become a common and important regulatory mechanism affecting its expression, especially ubiquitination. CSN6 promoted p16 degradation in gastric cancer via ubiquitination 15. UHRF1 promoted cell growth and metastasis by p16 repression in colorectal cancer 16. Similar to other tumours, low expression of p16 predicts poor prognosis in PCa 17. However, the mechanism by which p16 is regulated in PCa remains unclear.

Epithelial-mesenchymal transition (EMT), where cells lose epithelial features and acquire mesenchymal characteristics, is critical for primary tumour cells invade through tumour stroma and disseminate into circulation. Multiple signalling pathways have been identified to involve in EMT, and the role of WNT/β-catenin signalling in EMT is more prominent in a variety of cancers, including PCa 18. WNT/β-catenin signalling activation facilitates bone metastasis of PCa by promoting tumour cells dissemination, homing and dormancy 19. Abnormal ubiquitination of β-catenin is one of the mechanisms of WNT signalling activation. Several ubiquitin enzymes, including FBXW2, TRIM33 and UBE2S, are identified as regulators of β-catenin in cancers 20-22. However, it is still unclear whether and how β-catenin is regulated by ubiquitination in PCa.

Ubiquitination is the most important posttranslational modification in physiological and pathological processes. The fate of the ubiquitinated protein is depends on the type of ubiquitination. In eukaryotic cells, target proteins can be modified by seven lysine residues of ubiquitin (K6, K11, K27, K29, K33, K48 and K63), which regulate various protein functions, including degradation, localization, stability increase and signal transduction. UBE2S belongs to the E2 family of ubiquitin-conjugating enzymes and has been reported to mediate Lys11(K11)-linked polyubiquitin for substrate protein ubiquitination. Previous studies have found that UBE2S is highly expressed in several cancers and promotes tumorigenesis via invasion and cell cycle acceleration. In cervical cancer, UBE2S increased cancer cell invasion through EMT signalling activation and MMP-9 expression 23. In addition, UBE2S accelerated the cell cycle by ubiquitination of p27 to promote hepatocellular carcinoma development 24. However, the essential mechanism of UBE2S in PCa, especially bone metastasis, is not clearly understood.

In the present study, by sequencing PCa bone metastases, we found that an abnormal cell cycle was significantly associated with bone metastasis of PCa, while p16 posttranslational downregulation played an important role in this process. Next, we demonstrated that UBE2S degraded p16 and stabilized β-catenin through K11-linked ubiquitination. Moreover, we first revealed that UBE2S not only promoted tumour cell proliferation and invasion, but also predicted poor prognosis in PCa bone metastasis. Targeting UBE2S by cephalomannine is a promising therapy that inhibits bone metastasis and proliferation of PCa.

## Materials and Methods

### Patients and clinical samples

Cohort 1 included a total of 9 formalin-fixed, paraffin-embedded primary sites of metastatic PCa specimens and 27 bone metastases of prostate specimens from SYSMH between January 2007 and December 2018. Cohort 2 included a total of 106 formalin-fixed, paraffin-embedded localized PCa specimens and 43 normal adjacent prostate specimens from SYSUCC between January 2005 and December 2018. All samples were immunohistochemically stained and diagnosed by two pathologists.This project was approved by the Institutional Ethical Boards of Sun Yat-sen Memorial Hospital (SYSMH) (SYSEC-KY-KS-2020-201). The clinicopathological characteristics of Cohorts 1 and 2 are summarized in Supplementary Tables 2.

### RNA-seq

This study collected a total of 19 samples, including 7 cases of PCa primary sites without metastasis, 6 cases of primary sites of bone metastatic PCa and 6 cases of bone metastases of PCa, that underwent surgery or biopsy from Sun Yat-sen Memorial Hospital of Sun Yat-sen University (Guangzhou, China). All of the samples were immediately snap-frozen with liquid nitrogen and preserved at -80 °C until total RNA and protein were extracted. All samples were immunohistochemically stained and diagnosed by two pathologists. The clinicopathological characteristics of the 19 patients are summarized in Supplementary Table 1. RIBOBIO Gene Technology (Guangzhou, China) was used for library construction and sequencing. Genes with Log_2_(Fold Change) absolute value >1 and p value <0.05 in the comparison group were determined to be differentially expressed. All raw data of RNA sequencing (RNA-seq) have been uploaded to National Genomics Data Center (https://ngdc.cncb.ac.cn/) (HRA002360).

### Cell lines, cell culture and cell synchronization

PC3 and 22RV1 cells were obtained from the American Type Culture Collection (ATCC, Manassas, VA, USA). The human PCa cell lines PC-3M-IE8 and PC-3M-2B4 were obtained from Cell Resource Center, Peking Union Medical College (CRCPUMC, Beijing, China), which is the headquarters of National Infrastructure of Cell Line Resource (NSTI). PC-3M-IE8, PC-3M-2B4, PC3 and 22RV1 cells were cultured in RPMI 1640 (Gibco, Shanghai, China), which contained 10% foetal bovine serum (FBS, Sigma-Aldrich, USA) and 1% penicillin-streptomycin (Gibco, Shanghai, China). All cells were cultured in an incubator with 5% CO_2_ at 37 °C. All cell lines used in this study were excluded from mycoplasma contamination. Short tandem repeat (STR) authentication of the PCa cell lines PC-3 and 22RV1 was conducted to prove that there was no misidentification or contamination with other cells. STR authentication was conducted by IGE Biotechnology LTD, Guangzhou, China. The identities of the PC-3M-IE8 and PC-3M-2B4 cell lines were authenticated with STR profiling (FBI, CODIS). All the results of PC-3M-IE8 and PC-3M-2B4 can be viewed on the website (http://cellresource.cn). Cell synchronization by double thymidine block to arrest cell at G1/S boundary as previous reported 25.

### Immunohistochemistry (IHC)

IHC was performed on FFPE tissues as previously described 26. Briefly, the staining intensity was scored using four grades: negative (0, no staining), weak (1, light yellow), medium (2, brown) and strong (3, brown red). The percentage of tumour cells with positive staining was also divided into four grades: 0 (no positive), 25 (positive ≤ 10%), 50 (10% < positive ≤ 30%), 75 (30% < positive ≤ 70%), and 100 (positive >70%). The stain score (H-score) was calculated by multiplying the stain intensity and the percentage of tumour cells with positive staining, which ranged from 0 to 300. A H-score of UBE2S greater than 120 was defined as high expression, and vice versa.

### Oncomine and TCGA database mining

The expression of UBE2S in the Grasso prostate, Taylor prostate, Yu prostate, Holzbeierlein prostate, LaTulippe prostate and Ramaswamy prostate cohorts is available in the Oncomine database (www.oncomine.org). The clinical and expression profiles of TCGA-PRAD cohort are available at https://xenabrowser.net/datapages/. Excluding samples without UBE2S expression, T stage and N stage, we finally selected a cohort containing 52 normal and 491 tumours to analyse the prognosis of UBE2S. The clinicopathological characteristics of TCGA PRAD database are summarized in Supplementary Table 2.

### RNA interference

All siRNAs targeting UBE2S, p16 and the negative control were purchased from GenePhama (Shanghai, China) and were listed in Supplementary Table 6. As described in a previous study 27, Lipofectamine RNAiMAX (Thermo Fisher Scientific, USA) was used to transfect siRNAs according to the manufacturer's instructions.

### Construction of stable knockdown cell lines

The shRNA sequences of UBE2S were cloned into the pLKO.1-Puro vectors and were listed in Supplementary Table 6. The cloned plasmid was transfected into 293T cells to produce lentivirus. Furthermore, PC-3M-IE8 and 22RV1 cells were transfected with lentivirus and screened with puromycin. The specific steps of lentivirus production and infection were previously described 28.

### Cell proliferation assays

CCK8 assay and the colony formation assay were applied to detect cell viability. Cell cycle analysis was applied to detect cell populations at different cell cycle phases. All of the above experiments were performed as previously described 27.

### Transwell migration and invasion assays

The migration and invasion of PCa cells were evaluated by transwell assays. The penetrated cells were analysed and imaged by microscopy (Carl Zeiss Microscopy, Germany). All of the above experiments were performed as previously described 29.

### Real-time quantitative PCR

TRIzol (TaKaRa, Dalian, China) was used to extract total RNA from cells as previously described 30. RNA (1000 ng) was used for reverse transcription with the PrimerScript RT-PCR kit (TaKaRa). qPCR was performed on a LightCycler 480 system with a SYBR Green PCR kit (TaKaRa), and the 2-ΔΔCt method was used to calculate the relative levels of mRNA. All the primers used in this study are listed in Supplementary Table 7.

### Western blot

Western blots were conducted as previously described 31. The primary antibodies used in this study are listed in Supplementary Table 8. The PVDF membranes were incubated with secondary antibodies (anti-mouse or anti-rabbit, Proteintech, China) and visualized using Immobilon enhanced chemiluminescence (Millipore).

### Bone metastasis xenograft models

All animal procedures in this study were approved by the Sun Yat-sen University Institutional Animal Care and Use Committee, and the approval number is L102042021080M. Five-week-old male BALB/c nude mice were obtained from Shanghai SLAC Laboratory Animal Co., Ltd. The caudal arteries injecting xenograft murine models were constructed as previously reported 32. Briefly, for the UBE2S knockdown *in vivo* experiment, 2×10^6^ PC-3M-IE8-luc cells with or without UBE2S knockdown were injected into each mouse at a moderate rate through the caudal artery, and each group contained 6 mice. After tumour cell injection, bone metastases were monitored and captured every week using a bioluminescent imaging system (Bruker MI). For the *in vivo* experiment, 2×10^6^ PC-3M-IE8-luc were injected into each mouse at a moderate rate through the caudal artery. When the bioluminescence signal appeared (approximately 4-5 weeks), all mice with signals were divided into 3 groups, with 4 mice in each group. The control group was intraperitoneally injected with PBS, and the treatment group was intraperitoneally injected with cephalomannine (10 mg/kg and 20 mg/kg, respectively). Similarly, bone metastases were monitored and captured every week using a bioluminescent imaging system (Bruker MI). X-ray images were collected at an exposure of 10 sec and 35 keV. μCT was performed for the dissected hind legs from euthanized mice by Bruker skyscan1276. Finally, the dissected tissues were enucleated and embedded in paraffin. The sum of BLI signals of bone metastases in each nude mouse up to 400 was defined as an end point event.

### TRAP/ALP double staining

A TRAP/ALP Stain Kit (Wako, 294-67001) was used for staining. All steps followed the manufacturer's instructions. Briefly, paraffin sections were fully dewaxed, and TRAP, ALP and DAPI staining was added in sequence. TRAP and ALP were incubated at RT in a moist chamber for 30 minutes, and DAPI was incubated for 4-5 seconds. The sections were dried and photographed by microscopy (Carl Zeiss Microscopy, Germany).

### Illustration

A schematic model of the mechanism was created with BioRender.com. A Venn diagram was created with Venny (version 2.1, https://bioinfogp.cnb.csic.es/tools/venny). Reactome pathway analysis was performed with Kobas (http://bioinfo.org/kobas) 33, and a bubble chart was created using the ggplot2 package 34 in R software.

### Statistical analysis

All quantitative data are based on three independent experiments and presented as the mean±SD. For parametric variables, Student's t test or one-way analysis of variance (ANOVA) was used for group comparisons. For nonparametric variables, the chi-square test (χ^2^ test) was used for group comparisons. The correlation of UBE2S expression and clinicopathological characteristics in our cohort and TCGA PRAD data was analysed using Pearson's test. Kaplan-Meier analysis was used to analyse the OS and PFS of UBE2S. Cox proportional hazard models were used to analyse the effect of UBE2S on survival. All data analysis and chart drawing were performed by SPSS 24.0 and GraphPad Prism 8.0. A difference was considered statistically significant at *p <.05 and **p < 0.01.

## Results

### p16 controls the G1/S transition and plays an important role during PCa metastasis

To investigate the important process of PCa metastasis, we performed transcriptome sequencing of 7 cases of nonmetastatic primary PCa (primary site), 6 cases of metastatic PCa (mPCa) and 6 cases of bone metastasis (BM) of PCa. The patient characteristics are shown in Supplementary Table 1. As shown in Fig. S1 A and B, there were 1103 upregulated genes and 930 downregulated genes in the mPCa vs. primary site group. In addition, 1739 upregulated and 1916 downregulated genes were identified in the BM vs. primary site group (Fig. S1C & D). Given that oncogenes have become more likely to be targets for cancer therapy, we focused on the upregulated genes in bone metastasis of PCa in the hope of discovering potential therapeutic targets. Through pathway analysis of upregulated genes, the cell cycle, especially the G1/S phase pathway, was one of the most enriched pathways in both metastatic PCa and bone metastases compared with nonmetastatic primary PCa (Fig. S2A & B). This suggests that tumour cell proliferation may play a key role during PCa metastasis, particularly when tumour cells have been colonized and developed to bone metastases. Further analysis was performed to identify upregulated genes in the mPCa and BM groups compared to primary sites, and downstream genes of G1/S phase, such as E2F1, CDT1, RRM2 and CDC6, were overexpressed both in the mPCa and BM groups at the mRNA level (Fig. S3A). However, we found that the mRNA levels of key upstream genes associated with G1 phase, including p15, p16, p18, p19, p21, p27 and CCND1, were not significantly changed during PCa bone metastasis (Fig. S3B).

**Figure 1:**
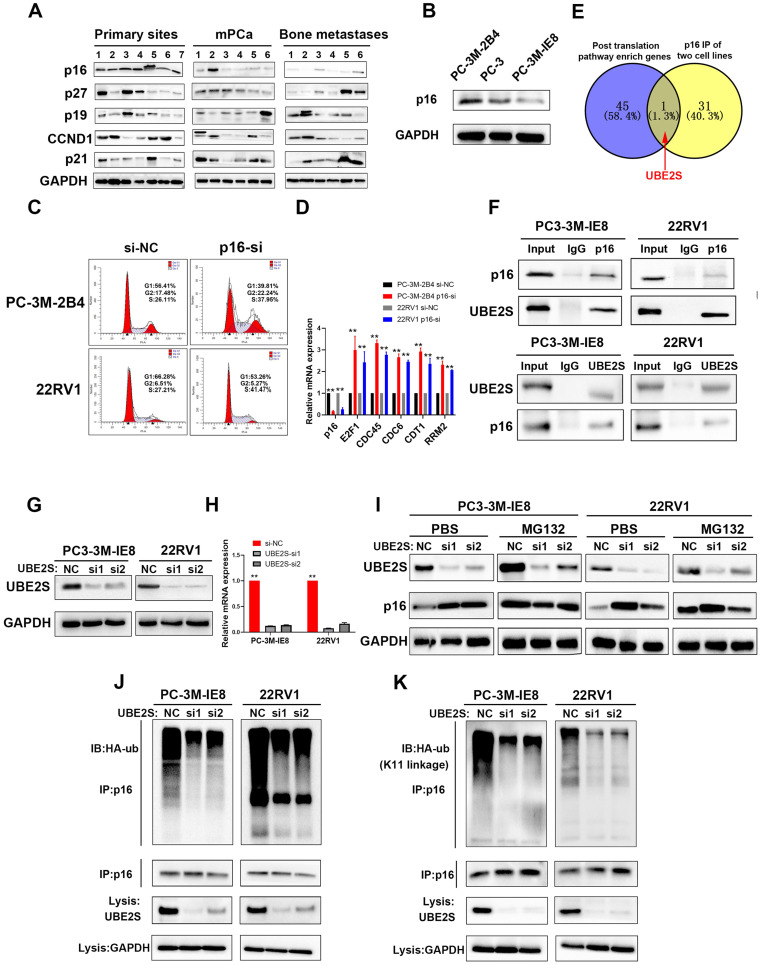
** p16 is downregulated in bone metastasis and degraded by UBE2S-mediated K11-linked ubiquitination in PCa. A.** Western blot (WB) analysis of p16, p27, p19, CCND1 and p21 in 19 sequencing PCa tissues. The amount of protein loaded and exposure time of all lanes are the same. **B.** WB analysis of p16 in PC-3M-2B4, PC-3, and PC-3M-IE8 cells. **C.** Representative images of cell cycle analysis using PC-3M-2B4 and 22RV1 cells treated as indicated. **D.** The downstream genes of the G1/S transition were verified in PC-3M-IE8 and 22RV1 cells by qRT-PCR. **E.** Intersection of posttranslation pathway-enriched genes and p16 IP of two cell lines. **F.** Coimmunoprecipitation (Co-IP) analysis shows the binding between endogenous UBE2S and endogenous p16 in G1/S boundary synchronized PC-3M-IE8 and 22RV1 cells. **G-H.** WB (g) and qPCR (h) analysis of UBE2S expression in PC-3M-IE8 and 22RV1 cells treated with or without UBE2S siRNA. **I.** WB analysis of p16 in PC-3M-IE8 and 22RV1 cells transfected with si-NC or si-UBE2S after MG132 (10 µM, 10 h) or PBS treatment. **J.** Co-IP analysis of ubiquitination of p16 in UBE2S-knockdown PC-3M-IE8 and 22RV1 cells transfected with HA-ub plasmid and synchronized in G1/S boundary. **K.** Co-IP analysis of ubiquitination of p16 in UBE2S-knockdown PC-3M-IE8 and 22RV1 cells transfected with the K11-linked HA-ub plasmid and synchronized in G1/S boundary. Statistical significance was assessed using a two-tailed t test or one-way ANOVA. Error bars represent the standard deviations of three independent experiments. *p < 0.05, **p < 0.01.

Next, we analysed the protein levels of the main regulators in G1/S phase, including p16, p19, p21, p27 and CCND1, in our 19 PCa tissues. As shown in Fig. 1A, the protein levels of p16, but not those of p19, p21, p27 and CCND1, were significantly lower in metastatic PCa and bone metastases. Furthermore, we obtained the high invasive potential PC3 subclone termed PC-3M-IE8 and the low invasive potential PC3 subclone termed PC-3M-2B4 from CRCPUMC 35,36 and validated their invasive potential *in vitro* as previously reported (Fig. S4A-E). Similarly, p16 expression gradually decreased from the low invasive PC3 subclone to the high invasive subclone (Fig. 1B). Silencing p16 in PC-3M-2B4 and 22RV1 cells with siRNA also proved that p16 knockdown increased the G1/S transition in PCa cells (Fig. 1C & S5) and upregulated the downstream genes at the mRNA level (Fig. 1D). Our results indicated that the G1/S phase pathway enriched in upregulated genes was induced by decreasing the protein level of p16 in PCa.

### UBE2S regulates the stability of p16 through K11-linked ubiquitination

We found that the protein level of p16 did not correspond to its mRNA level, suggesting posttranslational regulation of p16. To clarify this, we performed Reactome pathway analysis of 459 upregulated genes from the intersection of genes between the BM vs. primary sites group and the BM vs. mPCa group (Fig. S6A). As shown in Fig. S6B, posttranslational protein modification ranked eighth in the descending order of enrichment score, which suggested that p16 was posttranslationally regulated during PCa bone metastasis. Next, we performed coimmunoprecipitation (Co-IP) assays of p16 (Fig. S6C), followed by mass spectrometry in G1/S boundary synchronized PC-3M-IE8 and 22RV1 cell lines, and discovered 32 potential binding proteins. By intersecting the pathway enriched genes and mass spectrum of p16, we found that UBE2S was the only candidate meeting the conditions (Fig. 1E). The interaction between p16 and UBE2S was further validated by Co-IP and western blot (Fig. 1F & Fig. S6D). Moreover, UBE2S also had the highest mRNA expression level of the pathway-enriched genes, meaning that it was easier to achieve siRNA knockdown (Fig. S6E). To our knowledge, UBE2S, as a member of the ubiquitin-conjugating E2 enzyme, has been reported to bind to its cofactor CDC20 and mediate K11-linked ubiquitin chains on substrates 37. To evaluate whether p16 was regulated by UBE2S, we silenced UBE2S in PCa cells with 2 different siRNAs (Fig. 1G & H). The protein expression of p16 was significantly increased in UBE2S knockdown PCa cells but was mainly attenuated after treatment with MG132 for 12 h, suggesting that UBE2S might regulate the stability of p16 (Fig. 1I). Furthermore, silencing UBE2S significantly reduced the total ubiquitination of p16 in PCa cells (Fig. 1J). To further clarify the type of ubiquitination mediated by UBE2S, we transfected HA-K11-, HA-K48- and HA-K63-linked ubiquitin plasmids into G1/S boundary synchronized PCa cells with or without UBE2S silencing. Interestingly, UBE2S knockdown only decreased the K11-linked ubiquitination of p16 but not the K48-linked and K63-linked ubiquitination in PCa cells (Fig. 1K and S7A & B). Taken together, our results demonstrate that UBE2S regulates the stability of p16 through K11-linked ubiquitination.

### UBE2S enhances PCa cell proliferation *in vitro* and bone metastasis *in vivo*

**Figure 2:**
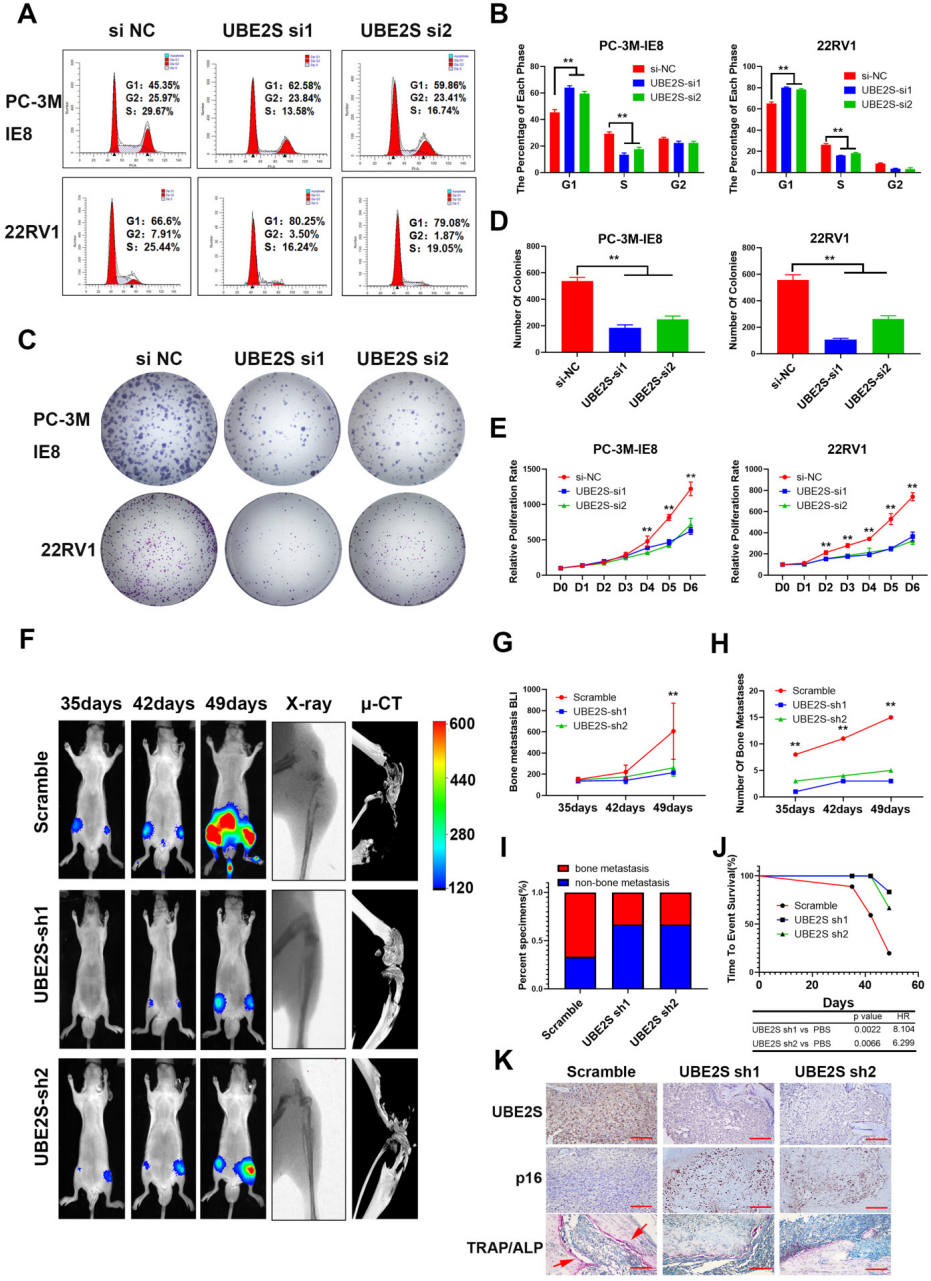
** UBE2S enhances PCa cell proliferation *in vitro* and bone metastasis *in vivo.* A-B**. Representative images (A) and histogram analysis (B) of cell cycle analysis using PC-3M-IE8 and 22RV1 cells treated as indicated. **C-D.** Representative images (C) and histogram analysis (D) of the colony formation assay using PC-3M-IE8 and 22RV1 cells treated as indicated. **E.** Effects of UBE2S on cell proliferation in PC-3M-IE8 and 22RV1 cells detected by CCK8 assay. **F.** Representative images of bioluminescence (BLI), X-ray and μ-CT of bone metastasis through caudal artery injection of the PC-3M-IE8/luc UBE2S-knockdown or PC-3M-IE8/luc scramble cell lines into nude mice. n= 6 mice/group, images representative of the median signal from (G). **G.** BLI quantification of bone metastasis in nude mice. **H.** Histogram analysis of the number of bone metastases in nude mice in each group. **I.** Percentage of bone metastasis status in all groups (n = 6). **J.** Kaplan-Meier curves for survival of mice in each group. **K.** Representative immunohistochemical images of UBE2S and p16 and images of TRAP/ALP double staining in each group. Red arrow: TRAP positive area, Scale bars: red, 50 µm. *p < 0.05, **p < 0.01.

To evaluate the biological roles of UBE2S in PCa, we first transfected two independent small interfering RNAs (siRNAs) to knockdown UBE2S in PCa cells. UBE2S knockdown dramatically increased the percentage of PCa cells in G0/G1 phase but reduced the percentage of PCa cells in S phase (Fig. 2A & B). Colony formation and CCK8 assays showed that silencing UBE2S significantly reduced the number of colonies formed and the rate of proliferation (Fig. 2C-E). Furthermore, the Annexin V/PI apoptotic assay was performed to estimate whether UBE2S is involved in the apoptosis of PCa, and it had no effect on apoptosis (Fig. S8).

In addition, we established shRNA-mediated stable cell lines by lentivirus infection. The biological function of UBE2S was further examined *in vivo* using a murine model of bone metastasis by caudal arteries injected with PC-3M-IE8/luc-sh-UBE2S and PC-3M-IE8/luc-sh-Ctrl (Fig. 2F). Bioluminescent imaging (BLI) analyses revealed that knockdown of UBE2S significantly reduced the intensity and number (Scramble 15 sites vs. sh1 3 sites vs. sh2 5 sites) of bone metastases (Fig. 2G-H). X-ray and micro-CT (μCT) analyses revealed that the bone lesion significantly decreased in the UBE2S silencing groups (Fig. 2F). Moreover, compared to the control group, the frequency of bone metastases also decreased in the PC-3M-IE8-sh-UBE2S group (scramble 67% vs. sh1 33% vs. sh2 33%) (Fig. 2I). UBE2S knockdown also significantly prolonged the time to arrival of the end point event (end point event was defined as the sum of BLI signals of bone metastases in each nude mouse up to 400) (Fig. 2J). Next, we dissected bone metastases and validated them via UBE2S and p16 staining. Obviously, the expression of UBE2S was lower in the sh-UBE2S group than in the scramble groups, but the expression of p16 was significantly increased in the sh-UBE2S group compared with the scramble groups (Fig. S9A & B). The expression of UBE2S and p16 was negatively correlated in nude mice (Fig. S9C). The marker enzymes of osteoclast and osteoblasts are tartrate-resistant acid phosphatase (TRAP) and alkaline phosphatase (ALP), respectively. In TRAP/ALP double staining, TRAP positive osteoclasts were red, and ALP positive osteoblasts were brown. The activity of osteoclasts and osteoblasts can be reflected by staining degree. TRAP staining in UBE2S knockdown group was significantly decreased compare with control group, indicating that UBE2S knockdown reduced osteoclasts activity and bone destruction (Fig. 2K). Collectively, these results demonstrate that UBE2S enhances tumour cell proliferation and facilitates bone metastasis of PCa *in vitro* and *in vivo*.

### The inhibition of p16 reverses the proliferation effects of UBE2S Knockdown

**Figure 3:**
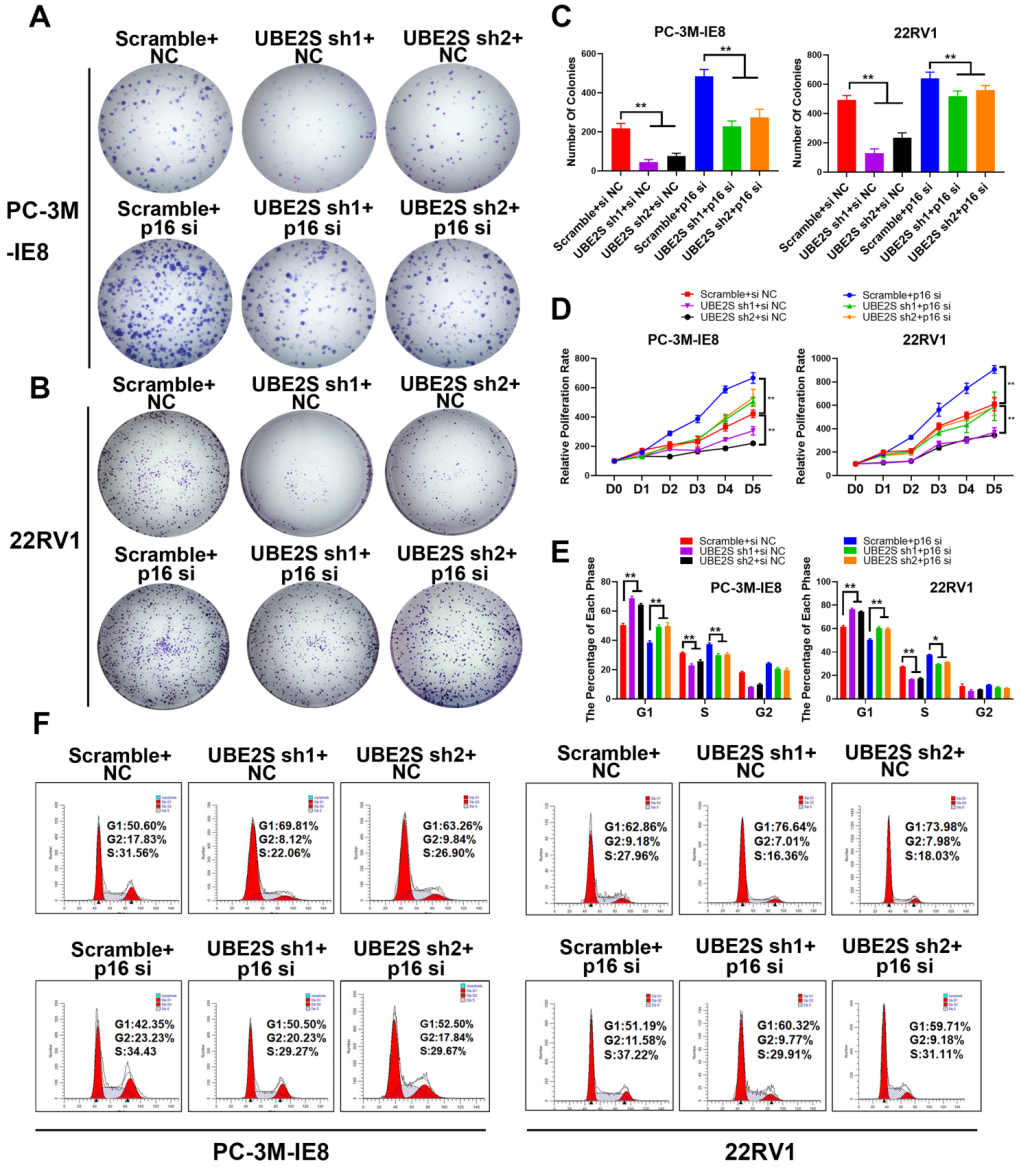
** The inhibition of p16 reverses the proliferation effects of UBE2S knockdown. A-C.** Representative images (A-B) and histogram analysis (C) of the colony formation assay using PC-3M-IE8 and 22RV1 cells treated as indicated. **D.** Effects of p16 on cell proliferation in PC-3M-IE8 and 22RV1 cells with UBE2S knockdown or not detected by CCK8 assay. **E-F.** Representative images (F) and histogram analysis (E) of cell cycle analysis using PC-3M-IE8 and 22RV1 cells treated as indicated. *p < 0.05, **p < 0.01.

To further clarify whether UBE2S regulates tumour cell proliferation of PCa by degrading p16, we silenced p16 in UBE2S-knockdown cells via siRNA. Consistent with our hypothesis, silencing p16 effectively reversed the inhibition of proliferation and clone formation ability in UBE2S-knockdown cells through CCK8 and colony formation assays (Fig. 3A-D). Furthermore, the percentage of UBE2S-knockdown cells in G0/G1 phase and S phase returned to a level similar to that in the control groups after p16 silencing (Fig. 3E & F). Taken together, these data reveal that UBE2S regulates the cell cycle of bone metastasis in PCa in a p16-dependent manner.

### UBE2S promotes metastasis by stabilizing β-catenin via K11-linked ubiquitination

**Figure 4:**
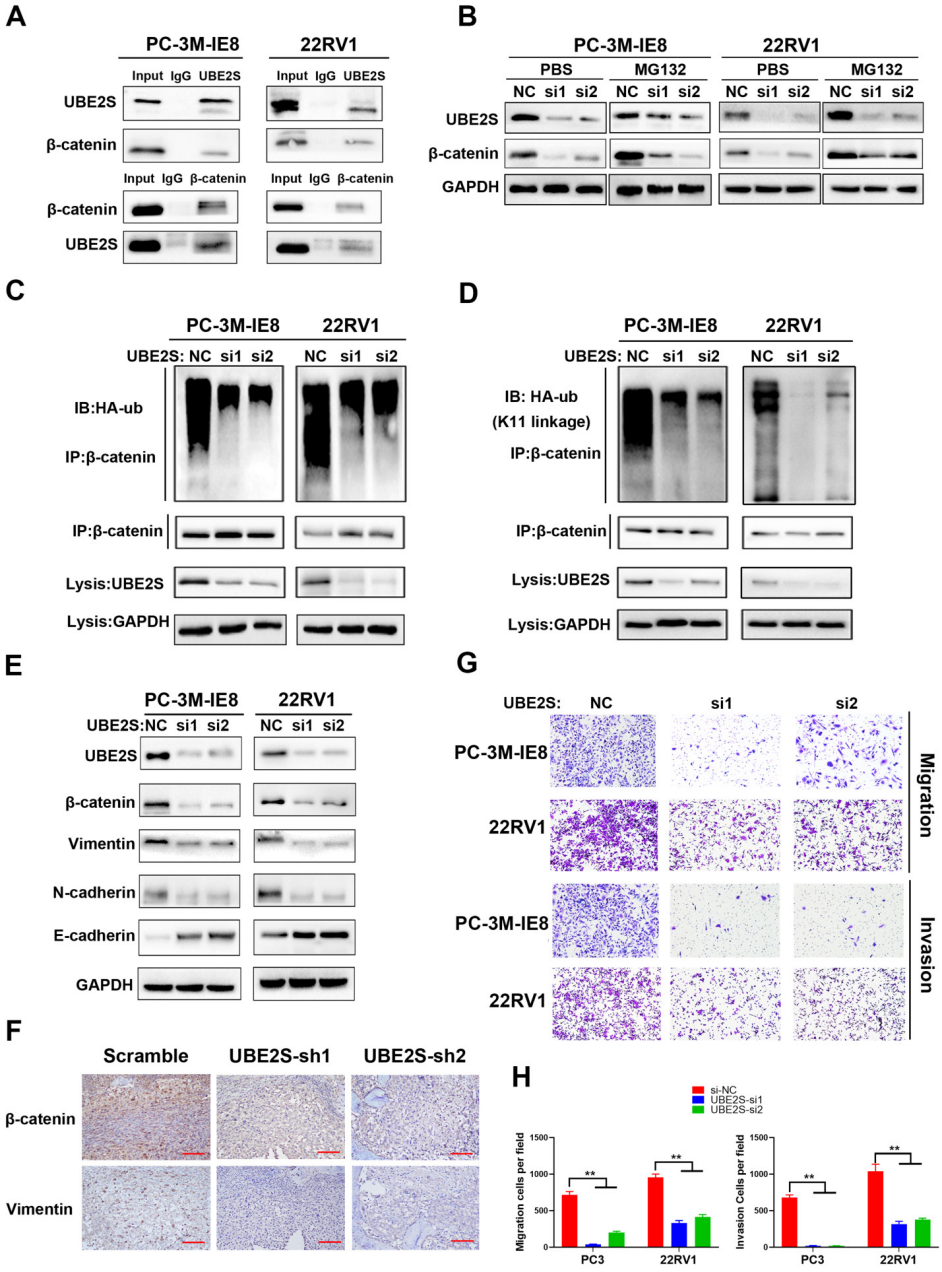
** UBE2S promotes metastasis by stabilizing β-catenin via K11-linked ubiquitination. A.** Co-IP analysis shows the binding between endogenous UBE2S and endogenous β-catenin in PC-3M-IE8 and 22RV1 cells. **B.** WB analysis of β-catenin in PC-3M-IE8 and 22RV1 cells transfected with si-NC or si-UBE2S after MG132 or PBS (10 µM, 10 h). **C.** Co-IP analysis of ubiquitination of β-catenin in UBE2S-knockdown PC-3M-IE8 and 22RV1 cells transfected with HA-ub plasmid. **D.** Co-IP analysis of ubiquitination of β-catenin in UBE2S-knockdown PC-3M-IE8 and 22RV1 cells transfected with the K11 linkage HA-ub plasmid. **E.** WB analysis of EMT signalling proteins in PC-3M-IE8 and 22RV1 cells transfected with the indicated siRNAs.** F.** Representative immunohistochemical images of β-catenin and vimentin in each group. Scale bars: red, 50 µm. **G-H.** Representative images (g) and histogram analysis (h) of migration and invasion assays using PC-3M-IE8 and 22RV1 cells treated as indicated. *p < 0.05, **p < 0.01.

Based on the bone metastasis assay *in vivo*, we found that UBE2S-knockdown cells not only had a smaller size of bone metastases but also fewer metastases and fewer metastasis ratios than control cells. This result strongly suggested that UBE2S regulated both the proliferation and invasion of PCa during bone metastasis. To address this, Co-IP assays of UBE2S followed by mass spectrometry (Fig. S10A & B) revealed that β-catenin was another potential binding protein of UBE2S and was further validated by western blotting (Fig. 4A). Interestingly, in contrast to the degradation of p16, UBE2S knockdown markedly reduced the expression of β-catenin but was not attenuated after treatment with MG132 for 12 h, suggesting that UBE2S stabilizes β-catenin during nonubiquitination degradation in PCa cells (Fig. 4B). Furthermore, UBE2S knockdown also reduced the total ubiquitination of β-catenin (Fig. 4C). Next, we transfected HA-K11-, HA-K48- and HA-K63-linked ubiquitin plasmids into PCa cells and performed Co-IP of β-catenin. Similar to p16, UBE2S increased K11-linked ubiquitination of β-catenin (Fig. 4D) but not K48- or K63-linked ubiquitination (Fig. S10C & D). β-catenin, as a product of the oncogene CTNNB1, has been reported to be involved in WNT signalling and plays a significant role in tumour cell EMT and cancer metastasis 19. Therefore, we detected the expression of epithelial and mesenchymal proteins as markers of EMT by western blotting. Compared to the control group, the expression of the mesenchymal markers N-cadherin and Vimentin was decreased, while the expression of the epithelial marker E-Cadherin was increased in the UBE2S silencing group in PCa cells (Fig. 4E). In our *in vivo* model, the expression of β-catenin and Vimentin in dissected bone metastases was significantly decreased in the UBE2S-silenced groups (Fig. 4F and S11A & B) and the expression of UBE2S was positively correlated with β-catenin and Vimentin expression (Fig. S11C & D). According to the above results, we inferred that UBE2S probably exerted metastasis-related biological functions. As expected, UBE2S silencing significantly reduced the migratory and invasive speed of PCa cells *in vitro* (Fig. 4G & H). In summary, UBE2S stabilized β-catenin through K11-linked ubiquitination and promoted tumour cell migration and invasion in PCa bone metastasis.

### UBE2S is overexpressed in human metastatic PCa tissue and correlates with poor prognosis in PCa

**Figure 5:**
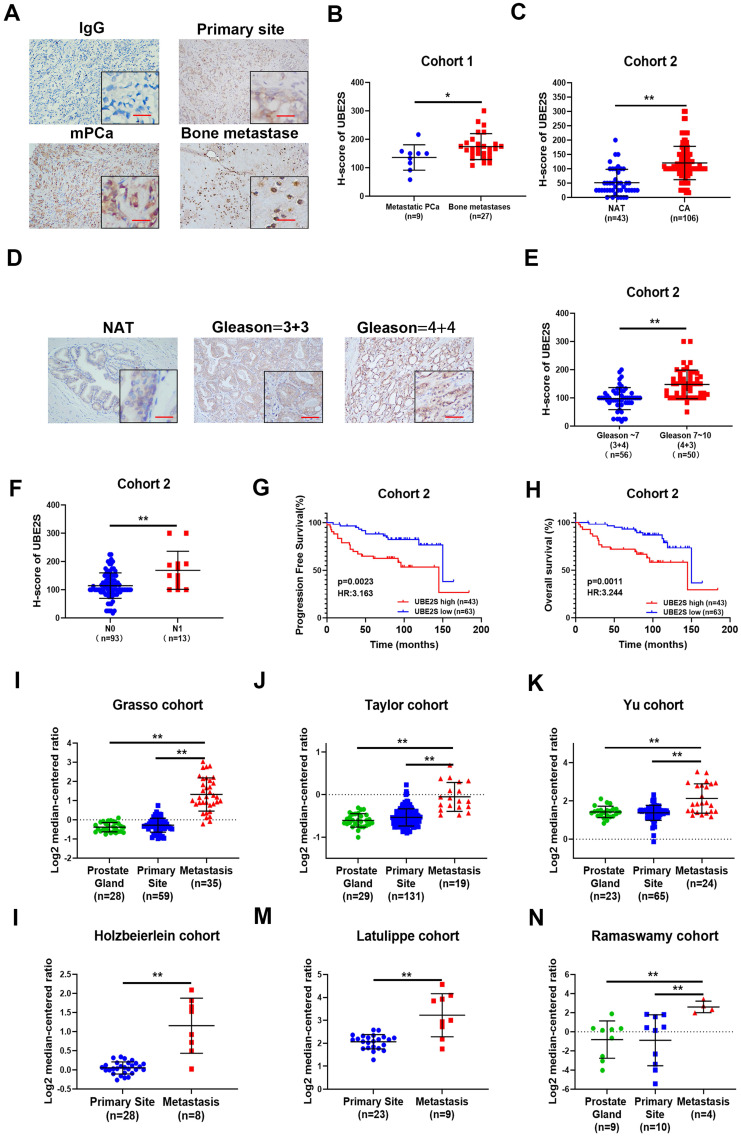
** UBE2S is overexpressed in human metastatic PCa tissue and correlates with poor prognosis in PCa. A.** Representative immunohistochemical staining images of UBE2S in the primary site of PCa, mPCa and bone metastasis of PCa. Scale bars: red, 50 µm. **B.** The expression difference of UBE2S between mPCa and bone metastasis of PCa in Cohort 1. **C.** The expression difference of UBE2S between NAT and PCa in Cohort 2.** D.** The expression difference of UBE2S between lymph node-negative and lymph node-positive PCa in Cohort 2. **E.** The expression difference of UBE2S between low Gleason score ((6-7(3+4)) PCa tissues and high Gleason score (7(4+3)-10) PCa tissues in Cohort 2. **F.** Representative immunohistochemical staining images of UBE2S in NAT and PCa with Gleason score = 3+3 and Gleason score = 4+4 in Cohort 2. Scale bars: red, 50 µm. **G-H.** Kaplan-Meier curves for progression free survival (g) and overall survival (h) of PCa patients with high or low expression of UBE2S in Cohort 2. **I-N.** The expression difference of UBE2S between prostate grand, primary site of PCa and mPCa in the Grasso, Taylor, Yu, Holzbeierlein, Latulippe and Ramasway cohorts. *p < 0.05, **p < 0.01.

To explore the clinical relevance of UBE2S in PCa, we detected its protein expression in the SYSMH cohort (Cohort 1) (including 36 bone metastatic PCa tissues) and SYSUCC cohort (Cohort 2) (including 106 PCa tissues and 40 nontumour adjacent tissues) by immunohistochemistry. In cohort 1, UBE2S expression was significantly upregulated in bone metastases of PCa compared with the primary site of metastatic PCa (Fig. 5A & B). In cohort 2, UBE2S expression was higher in PCa tissues than in normal adjacent tissues (NATs), and was especially higher in tissues with a Gleason score >7 (Fig. 5C-E). In addition, the expression of UBE2S was also significantly higher in PCa tissues with nodal metastasis than in those without nodal metastasis (Fig. 5F). Further Kaplan-Meier analysis revealed that patients with high protein levels of UBE2S had shorter progression free survival (PFS) and overall survival (OS) than those with low UBE2S levels in cohort 2 (Fig. 5G & H). To further validate our results, we analysed mRNA levels in the Oncomine database and TCGA database. Six studies, including the Grasso, Taylor, Yu, Holzbeierlein, LaTulippe and Ramaswamy cohorts in the Oncomine database, showed that UBE2S was expressed at higher levels in metastases than at the primary site in PCa (Fig. 5I-N). In TCGA PRAD database, UBE2S expression was upregulated in tumour tissue compared with adjacent non-tumor tissue and increased in higher Gleason score (Fig. S12A-B). However, in TCGA PRAD database, UBE2S expression was not associated with nodal metastasis status (Fig. S12C). Similar to cohort 2, upregulated expression of UBE2S in TCGA was associated with poorer PFS (Fig. S12D). Moreover, the clinicopathological parameters of UBE2S in TCGA and our cohort are shown in Supplementary Table 2. Univariate and multivariate Cox regression analyses demonstrated that high expression of UBE2S was an independent prognostic factor for PFS in cohort 2 and TCGA and for OS in cohort 2 (Supplementary Table 3-4). These results show that UBE2S positively correlates with advanced stage and predicts poor prognosis in metastatic PCa.

### Targeting UBE2S with cephalomannine inhibits proliferation and invasion *in vitro* and PCa cell bone metastasis* in vivo*

**Figure 6:**
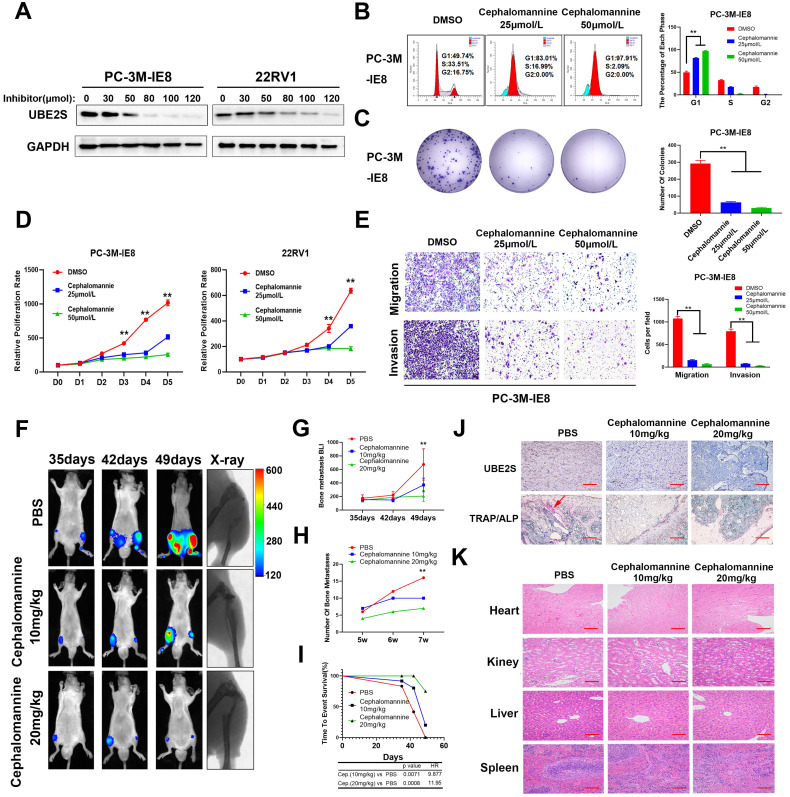
**Targeting UBE2S with cephalomannine inhibits proliferation and invasion *in vitro* and PCa cell bone metastasis *in vivo.* A.** WB analysis of UBE2S in PC-3M-IE8 and 22RV1 cells treated with varying concentrations of cephalomannine for 48 h. **B.** Representative images and histogram analysis of cell cycle progression in PC-3M-IE8 cells treated with varying concentrations of cephalomannine. **C.** Representative images and histogram analysis of the colony formation assay of PC-3M-IE8 cells treated with varying concentrations of cephalomannine. **D.** Effects of varying concentrations of cephalomannine on cell proliferation in PC-3M-IE8 and 22RV1 cells, as detected by CCK8 assay. **E.** Representative images and histogram analysis of migration and invasion assays of PC-3M-IE8 cells treated with varying concentrations of cephalomannine. **F.** Representative images of bioluminescence (BLI) and X-ray of bone metastasis through caudal artery injection of the PC-3M-IE8/luc cells into nude mice and treatment with varying concentrations of cephalomannine. n= 4 mice/group, images representative of the median signal from (H). **G.** BLI quantification of bone metastasis in nude mice treated with varying concentrations of cephalomannine. **H.** Histogram analysis of the number of bone metastases in nude mice treated with varying concentrations of cephalomannine. **I.** Kaplan-Meier curves for survival of mice in each group treated with varying concentrations of cephalomannine.** J.** Representative immunohistochemical images of UBE2S and images of TRAP/ALP double staining in varying cephalomannine concentration treatment groups. Red arrow: TRAP positive area, Scale bars: red, 50 µm. **K.** Representative H&E staining images of the heart, kidney, liver and spleen in the varying cephalomannine concentration treatment groups. Scale bars: red, 50 µm. *p < 0.05, **p < 0.01.

Next, we explored the effects of cephalomannine, which has been reported to inhibit UBE2S expression in hepatocellular carcinoma 24. The IC_50_ of cephalomannine was measured in PC-3M-IE8 and 22RV1 cells (Fig. S13A & B). The expression of UBE2S was gradually decreased with increasing cephalomannine concentration in PCa cells (Fig. 6A). CCK8, colony formation assay and cell cycle analysis provided evidence that cephalomannine inhibited tumour cell proliferation and arrested G1/S progression in PC-3M-IE8 (Fig. 6B-D) and 22RV1 (Fig. S13C-E) cells in a dose-dependent manner. In addition, cephalomannine also inhibited migration and invasion in both PC-3M-IE8 (Fig. 6E) and 22RV1 (Fig. S13F-G) cells in a dose-dependent manner. Next, we injected PC-3M-IE8/luc cells into the caudal arteries of nude mice. Four weeks after injection, the nude mice that had bone metastases were randomly divided into three groups and treated with PBS, 10 mg/kg or 20 mg/kg cephalomannine. As shown in Fig. 6F, X-ray analysis revealed that bone destruction decreased in the cephalomannine treatment groups compared to the control group. Both the BLI intensity (Fig. 6G) and number of bone metastases (Fig. 6H) were significantly reduced by cephalomannine treatment in a dose-dependent manner. Moreover, cephalomannine treatment significantly prolonged the time to arrival of the end point event (Fig. 6I). Immunochemistry showed that the expression of UBE2S was lower in the cephalomannine treatment group than in the control group (Fig. 6J). Consistently, TRAP/ALP double staining revealed that the number of osteoclasts in the treatment group was remarkably decreased, suggesting that bone destruction in the treatment group was suppressed (Fig. 6J). Furthermore, there was no apparent toxicity to the heart, liver or kidney in nude mice in the inhibitor groups (Fig. 6K). Blood analysis of nude mice in the inhibitor group also found no abnormalities in WBC, haemoglobin or liver and kidney functions (Supplementary Table 5). Collectively, these data demonstrate that cephalomannine dose-dependently inhibits UBE2S expression and thus suppresses PCa growth and metastasis *in vitro* and* in vivo*.

## Discussion

Bone metastasis is the most common metastasis and the main cause of poor prognosis in PCa 38. Although the mechanism of bone-specific metastasis has not been fully clarified, enhanced proliferation and invasion are necessary characteristics of tumour cells during bone metastasis. In our study, through RNA sequencing of primary nonmetastatic PCa, metastatic PCa and bone metastases of PCa tissues, we identified that one of the most important characteristics of PCa cells during bone metastasis was the G1/S transition, which was caused by the downregulation of p16. Next, we found that an ubiquitin-conjugating E2 enzyme termed UBE2S degraded p16 by K11-linked ubiquitination and promoted tumour cell proliferation. Furthermore, UBE2S enhanced EMT and invasion by stabilizing K11-linked ubiquitination of β-catenin. Targeting UBE2S with cephalomannine is a promising therapy that inhibits bone metastasis and proliferation of PCa.

UBE2S, as an E2-conjugating enzyme, has been reported to be upregulated in several cancers and to promote tumour development via ubiquitination. However, the role of UBE2S and ubiquitination in PCa is still unclear. In our study, we revealed that UBE2S enhanced PCa cell proliferation *in vitro* and bone metastasis *in vivo*. In addition, previous study has reported that low levels of p16 was associated with higher risk of distant metastases in PCa 39. In many cancers, point mutation, homozygous deletion and promoter hyper-methylation are the main causes of p16 inactivation. However, the frequency of mutation and deletion is not high in neither prostate cancer nor metastatic prostate cancer in public databases (Fig. S14A) 40,41. Furthermore, there was no significant difference in promoter methylation level between NAT and tumours as well as between different N-stage tumours in TCGA-PRAD (Fig. S14B & C). Recently, it has been reported that posttranslational modification of p16 is one of the important regulatory mechanism of p16 inactivation. In gastric cancer, CSN6 degrades p16 and promotes tumour progression through ubiquitination 15. However, it remains unclear whether low level of p16 is mediated by abnormal posttranslational modifications in PCa. In this study, we supplemented the mechanism that UBE2S physically bound to p16 and mediated its proteasomal degradation via K11-linked ubiquitination rather than K48- or K63-linked ubiquitination in PCa. Consistent with this, UBE2S has been recognized as the specific E2-conjugating enzyme that elongates Lys11-linked polyubiquitin chains on substrates and is involved in the cell cycle, signal transduction and tumour metastasis 42,43. Furthermore, our data showed that silencing p16 effectively reversed the inhibition of proliferation in UBE2S-knockdown cells, suggesting that UBE2S regulates the cell cycle in PCa in a p16-dependent manner. Taken together, UBE2S degraded p16 by K11-linked ubiquitination to promote tumorigenesis and bone metastasis in PCa.

Additionally, we further clarified that UBE2S enhanced the invasion of PCa cells* in vitro* and bone metastasis *in vivo*. Mechanistically, UBE2S also mediated K11-linked ubiquitination of β-catenin to increase its stability and therefore promoted tumour cell migration and invasion. Especially in bone metastasis, WNT/β-catenin signalling regulates tumour cell EMT, colonization and homing in a canonical or noncanonical manner 44-46. Interestingly, Zhang et al. reported that UBE2S modified and stabilized β-catenin through K11-linked ubiquitination in colorectal cancer, which is similar to what we found in PCa 47. Mechanistically, K11-linked ubiquitination of β-catenin at K19 mediated by UBE2S abolished the interaction between β-catenin and β-TrCP, which ultimately reduced S33/S37/S45/T41 phosphorylation of β-catenin and repressed β-TrCP-mediated degradation of β-Catenin. Although both p16 and β-catenin were ubiquitinated by UBE2S, their fates were quite different. This is probably due to the different E3 ligase enzymes binding with UBE2S and having opposite effects on substrates, which requires further research.

Here, we demonstrated that UBE2S was expressed at the highest level in bone metastases of PCa and gradually decreased in metastatic and nonmetastatic PCa and NAT. High expression of UBE2S was positively correlated with malignant grade and poor survival of PCa. Further analysis of the Oncomine database and TCGA database also supported that UBE2S was upregulated in metastatic PCa in six studies and correlated with poor PFS in TCGA. Consistent with our data, UBE2S was also overexpressed and predicted poor prognosis in glioma and endometrial cancer 48,49. In conclusion, high expression of UBE2S in PCa bone metastasis is associated with advanced metastatic status and poor prognosis, suggesting that UBE2S can be a biomarker of PCa bone metastasis and prognosis.

Compared to siRNA, targeting genes *in vivo* by small molecular inhibitors is more acceptable in many cancers, owing to its high selectivity and cell permeability. In a previous study, cephalomannine, as a natural compound, was reported to inhibit UBE2S in hepatocellular carcinoma (HCC) in a dose-dependent manner by suppressing its promoter activity. In our study, we discovered that cephalomannine also inhibited UBE2S expression and induced G1 phase arrest as well as impaired migration and invasion in PCa cells. Similarly, cephalomannine significantly inhibited tumour cell proliferation *in vitro* and* in vivo* via G1/S phase arrest in HCC cells, but the IC_50_ concentration of PCa cells was not consistent with that of HCC cells. Moreover, by constructing a caudal artery injection model in nude mice, we first discovered that cephalomannine dose-dependently reduced the size and number of bone metastases *in vivo*. Compared with the control groups, the cephalomannine treatment groups had significantly less bone destruction of the hind legs and smaller metastases. The nude mice in the inhibitor groups had no apparent toxicity to the heart, liver or kidney. However, the exact mechanism and pharmacokinetics of Cephalomannine should be further explored. Collectively, these results demonstrate that targeting UBE2S with cephalomannine may be a promising small molecular inhibitor to treat metastatic PCa since it inhibits tumour cell proliferation and invasion.

In summary, it is our novel discovery that PCa bone metastasis-related UBE2S enhances the cell cycle process and maintains EMT signalling activation by degrading p16 and stabilizing β-catenin in a K11-linked ubiquitination-dependent manner. Targeting UBE2S by cephalomannine might be a multipotent anticancer therapy and could offer novel therapeutic schemes against PCa metastasis.

## Supplementary Material

Supplementary figures and tables.

## Abbreviations

PCa: Prostate cancer
UBE2S: ubiquitin conjugating enzyme E2 S
RANK: TNF receptor superfamily member 11a
RANKL: TNF Superfamily Member 11
ub: ubiquitin
TRAP: Acid Phosphatase 5, Tartrate Resistant
ALP: Alkaline Phosphatase
qPCR: quantitative polymerase chain reaction
STR: Short Tandem Repeat
IC_50_: half maximal inhibitory concentration
CIs: confidence intervals
TCGA: The Cancer Genome Atlas
PFS: progression free survival
OS: overall survival
