# Supplementary material for: UBE2S as a novel ubiquitinated regulator of p16 and β-catenin to promote bone metastasis of prostate cancer
Source: Int J Biol Sci. 2022 May 16;18(8):3528–43. doi: 10.7150/ijbs.72629 (PMC9134922; doi:10.7150/ijbs.72629)
Supplement: Supplementary file 1 — Supplementary figures and tables. [file ijbsv18p3528s1.pdf]

Supplementary Table 1. Patient characteristics for RNA-sequencing.

Patient	Age	TNM stage	Gleason score	Metastase Therapy
Primary site 1	69	T3aN0M0	3+4	-
Primary site 2	69	T3bN0M0	4+4	-
Primary site 3	68	T3bN0M0	4+4	-
Primary site 4	73	T3aN0M0	4+3	-
Primary site 5	80	T3aN0M0	4+4	-
Primary site 6	68	T3aN0M0	4+5	-
Primary site 7	57	T3bN0M0	4+3	-
Metastatic PCa 1	74	T4N1M1b	4+4	No
Metastatic PCa 2	73	T4N1M1b	4+5	No
Metastatic PCa 3	70	T4N0M1b	4+4	Zoledronic acid & Surgery
Metastatic PCa 4	74	T4N0M1b	4+5	No
Metastatic PCa 5	68	T4N1M1b	4+4	Surgery
Metastatic PCa 6	65	T4N0M1b	4+5	Zoledronic acid
Bone metastasis 1	70	T4N0M1b	4+5	Surgery
Bone metastasis 2	87	T4N1M1b	4+4	Surgery
Bone metastasis 3	66	T3aN1M1b	4+3	Surgery
Bone metastasis 4	74	T3bN1M1b	4+4	Surgery
Bone metastasis 5	74	T3bN0M1b	4+5	Surgery
Bone metastasis 6	62	T4N1M1b	5+4	Surgery

Supplementary Table 2. UBE2S expression and clinicopathologic characteristics of prostate cancer patients in TCGA, Cohort1 and Cohort 2.

Variables		TCGA	Cohort1	Cohort 2
		Number of cases (%)	Number of cases (%)	Number of cases (%)
Age	< 60	200(40.7%)	6(16.7%)	26(24.5%)
	≥60	291(59.3%)	30(83.3%)	80(75.5%)
Gleason score	≤3+4	188(38.3%)	1(2.8%)	56(52.8%)
	≥4+3	247(50.3%)	12(33.3%)	50(47.2%)
T stage	T2	187(38.1%)	-	60(56.6%)
	T3	294(59.9%)	-	31(29.2%)
	T4	11(2.2%)	-	15(14.2%)
N status	Nx	70(14.3%)	-	0(0%)
	N0	341(69.5%)	-	93(87.7%)
	N1	80(16.3%)	-	13(12.3%)
UBE2S	Low	276(56.2%)	18(50%)	43(40.6%)
	High	215(43.8)	18(50%)	63(59.4%)

Supplementary Table 3. Univariate and multivariate Cox regression analyses of disease-free survival (DFS) in TCGA and Cohort 2.

Variables	TCGA				Cohort 2			
	Univariate		Multivariate		Univariate		Multivariate	
	HR (95%CI)	p-value	HR (95%CI)	p-value	HR (95%CI)	p-value	HR (95%CI)	p-value
**Age(y)** ≥60/ < 60	0.718 0.470-1.099	0.127	1.191 0.750-1.890	0.459	0.978 0.434-2.205	0.958	0.851 0.372-1.951	0.704
**Gleason** 7(4+3)-10/6-7(3+4)	4.257 2.368-7.654	**< 0.001****	3.117 1.507-6.446	**0.002****	3.047 1.399-6.637	**0.005****	1.662 0.663-4.166	0.279
**T stage** T3-4/T2	3.719 2.103-6.578	**< 0.001****	2.452 1.262-4.762	**0.008****	1.878 0.900-3.922	0.093	1.166 0.525-2.589	0.706
**N stage** N1/N0	1.868 1.154-3.023	**0.011****	1.193 0.721-1.973	0.493	3.512 1.598-7.722	**0.002****	3.172 1.442-6.974	**0.004****
**UBE2S** High/Low	2.265 1.483-3.461	**0.011****	1.891 1.204-2.970	**0.006****	2.932 1.418-6.063	**0.004****	2.773 1.328-5.791	**0.007****

Abbreviations: HR = hazard ratio; 95% CI = 95% confidence interval; T stage = tumor stage; N grade = lymph node grade; *p < 0.05, **p < 0.01.

Supplementary Table 4. Univariate and multivariate Cox regression analyses of overall survival (OS) in Cohort 2.

Variables	Cohort 2			
	Univariate		Multivariate	
	HR (95%CI)	p-value	HR (95%CI)	p-value
**Age(y)**	1.479	0.403	1.400	0.482
≥60/ < 60	0.592-3.695		0.548-3.577	
**Gleason**	2.952	**0.010***	1.239	0.676
7(4+3)-10/6-7(3+4)	1.297-6.718		0.454-3.379	
**T stage**	2.093	0.066	1.155	0.751
T3-4/T2	0.952-4.602		0.475-2.810	
**N stage**	4.614	**< 0.001****	4.631	**< 0.001****
N1/N0	2.050-10.384		2.050-10.460	
**UBE2S**	2.986	**0.005****	3.070	**0.005****
High/Low	1.386-6.434		1.398-6.743	

Abbreviations: HR = hazard ratio; 95% CI = 95% confidence interval; T stage = tumor stage; N grade = lymph node grade; *p < 0.05, **p < 0.01.

Supplementary Table 5. Effects of Cephalomannie on blood *in vivo*.

Blood Index	PBS (Mean±SD)	Cephalomannie 10mg/kg (Mean±SD)	Cephalomannie 20mg/kg (Mean±SD)	reference value
WBC	4.43±1.07(10^9/L)	2,33±0.83(10^9/L)	5.2±2.63(10^9/L)	0.8-6.8(10^9/L)
Granulocyte%	29.4±3.37(%)	23.07±5.27(%)	30.6±3.95(%)	8.6-38.9(%)
HGB	146.67±16.66(g/L)	115±19.95(g/L)	110±13.14(g/L)	110-143(g/L)
ALT	54.73±1.24(U/L)	40.60±3.68(U/L)	44.26±2.09(U/L)	10.06-96.47(U/L)
AST	163.50±6.46(U/L)	147.78±8.54(U/L)	194.79±24.50(U/L)	36.31-235.48(U/L)
ALB	30.48±0.47(g/L)	30.26±0.72(g/L)	31.54±1.54(g/L)	21.22-39.15(g/L)
ALP	142.83±16.44(U/L)	104.93±7.16(U/L)	116.08±12.76(U/L)	22.52-474.35(U/L)
CREA	18.35±0.83(μmol/L)	19.43±1.22(μmol/L)	25.30±6.21(μmol/L)	10.91-5.09(μmol/L)

Abbreviations: WBC= white blood cell count; HGB= hemoglobin; ALT= alanine transaminase; AST= aspartate aminotransferase; ALB= Albumin; ALP= alkaline phosphatase; CREA= Creatinine

Supplementary Table 6. Sequences of siRNA oligos and shRNAs used in this study.

Name	Sequence 5'-3'
**siRNA**	
si-Ctrl	UUCUCCGAACGUGUCACGUTT
si-UBE2S-1	ACAUCAUCCGCCUGGUGUATT
si-UBE2S-2	CAUAUGCUGGAGGUCUGUUTT
si-p16	CCCAACGCACCGAAUAGUUTT
**shRNA**	
sh-Ctrl	CAACAAGATGAAGAGCACCAA
UBE2S-sh1	ACATCATCCGCCTGGTGTA
UBE2S-sh2	CATATGCTGGAGGTCTGTT

Supplementary Table 7. Primers used in this study.

Primer name	Forward primer (5'-3')	Reverse primer (5'-3')	Application
UBE2S	ACAAGGAGGTGACGACACTGA	CCACGTTCGGGTGGAAGAT	qPCR
p16	ATGGAGCCTTCGGCTGACT	GTAACTATTCGGTGCGTTGGG	qPCR
E2F1	ACGTGACGTGTCAGGACCT	GATCGGGCCTTGTTTGCTCTT	qPCR
CDC45	TTCGTGTCCGATTTCCGCAAA	TGGAACCAGCGTATATTGCAC	qPCR
CDC6	CCAGGCACAGGCTACAATCAG	AACAGGTTACGGTTTGGACATT	qPCR
CDT1	CGGTGGACGAGGTTTCCAG	CTGCCGGGGTGGATTTCTT	qPCR
RRM2	GTGGAGCGATTTAGCCAAGAA	CACAAGGCATCGTTTCAATGG	qPCR
GAPDH	GTCTCCTCTGACTTCAACAGCG	ACCACCCTGTTGCTGTAGCCAA	qPCR

Supplementary Table 8. Antibodies used in this study.

Antibody Name	Cat No.	Brand
UBE2S	14115-1-AP	Proteintech
N-cadherin	22018-1-AP	Proteintech
E-cadherin	20874-1-AP	Proteintech
Beta catenin	51067-2-AP	Proteintech
GAPDH	60004-1-Ig	Proteintech
CCND1	60186-1-Ig	Proteintech
Vimentin	ab92547	Abcam
p16	A0262	ABclonal
p21	A19094	ABclonal
HA	AE008	ABclonal
p27	YP0201	Immunoway
p19	YT3496	Immunoway

Figure legend

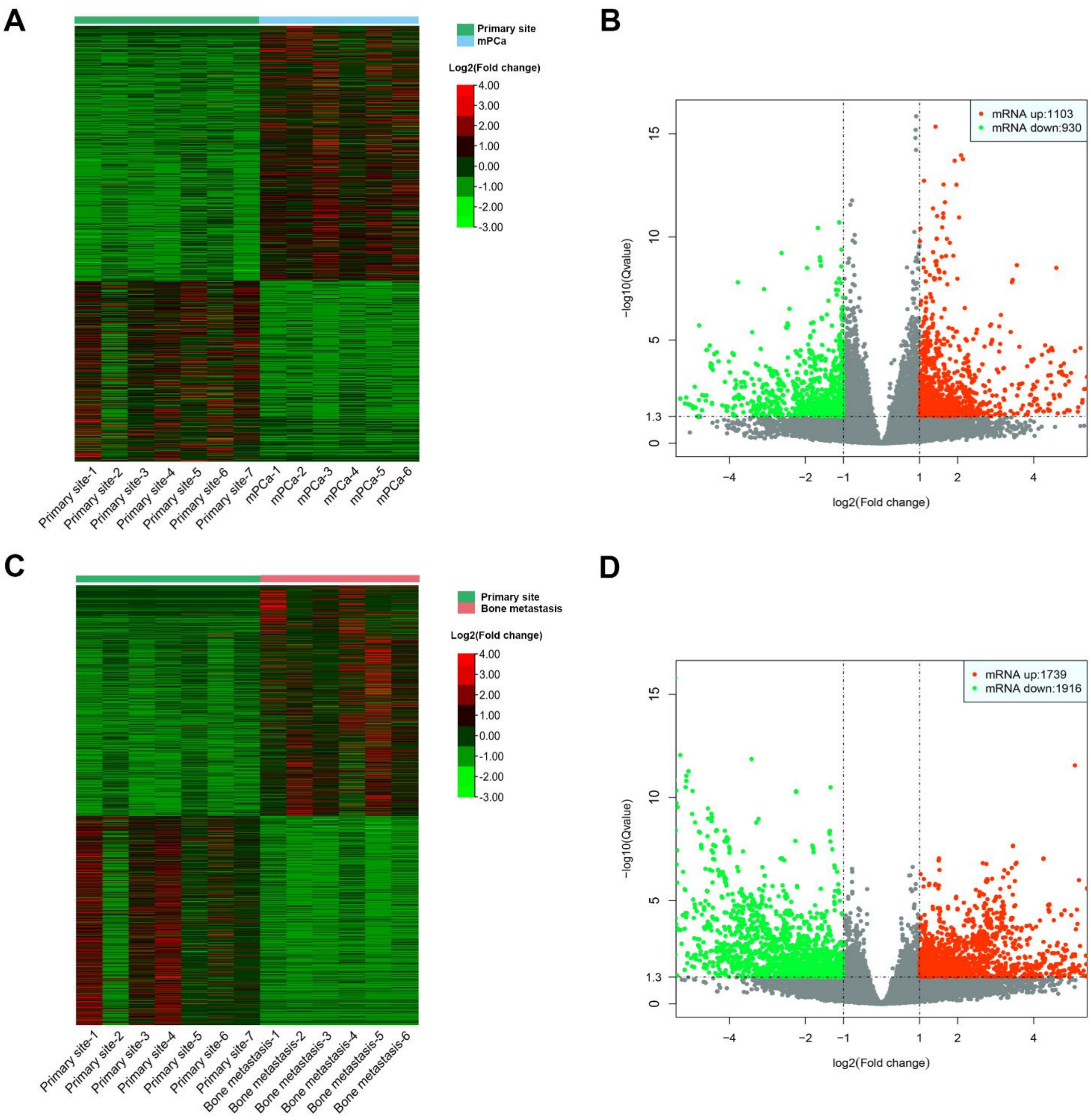

Supplemental Fig. 1 The heatmap and volcano of RNA-seq data in each group.

A-B The heatmap and volcano of mPCa vs Primary site group. **C-D** The heatmap and volcano of Bone metastasis (BM) vs Primary site group.

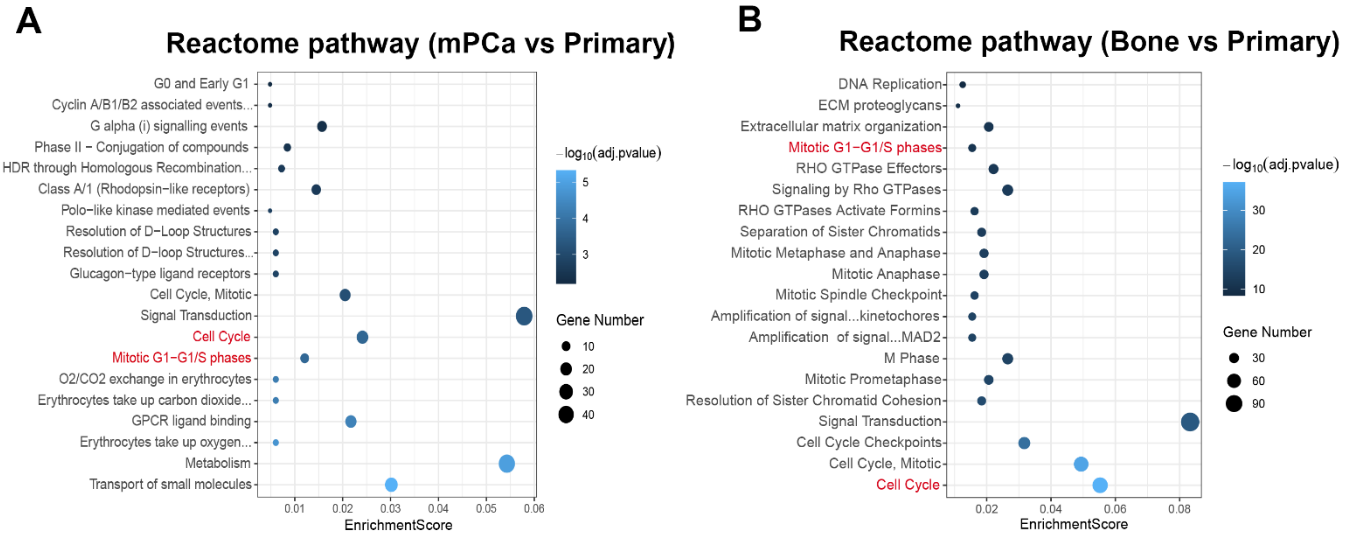

Supplemental Fig. 2 The Reactome pathway analysis of upregulated genes in each group.

A Bubble chart of Reactome pathway analysis of upregulated genes in mPCa vs Primary site group. **B** Bubble chart of Reactome pathway analysis of upregulated genes in BM vs Primary site group.

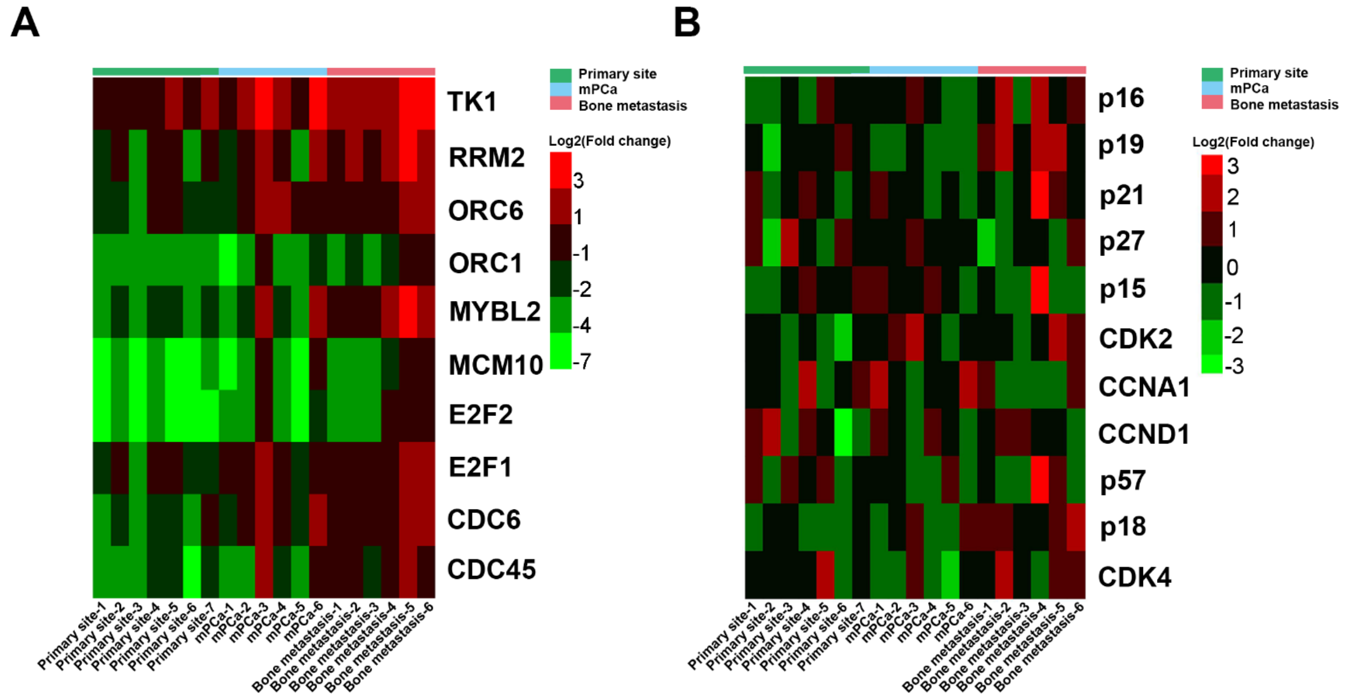

Supplemental Fig. 3 Heatmap of G1/S transition related genes.

A Heatmap of differentiated downstream genes in G1/S transition in each group. **B** Heatmap of key upstream regulators in G1/S transition in each group.

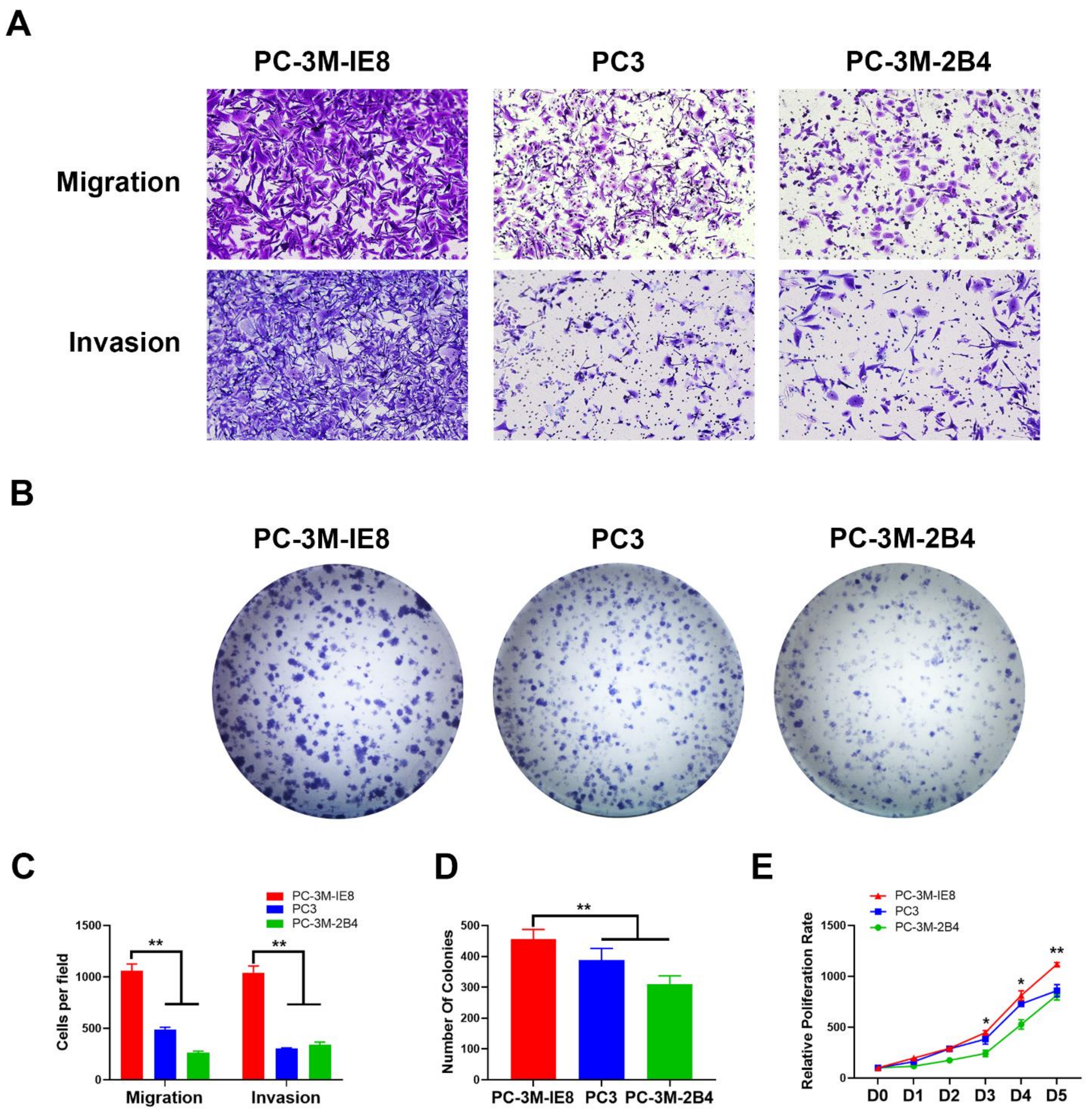

Supplemental Fig. 4 The invasive potential of PC-3M-2B4, PC-3 and PC-3M-IE8 *in vitro*

A Representative images of migration and invasion assays using PC-3M-2B4, PC-3 and PC-3M-IE8. **B** Representative images of colony formation assay using PC-3M-2B4, PC-3 and PC-3M-IE8. **C** Histogram analysis of migration and invasion assays using PC-3M-2B4, PC-3 and PC-3M-IE8. **D** Histogram analysis of colony formation assay using PC-3M-2B4, PC-3 and PC-3M-IE8. **E** Difference of cell proliferation in PC-3M-2B4, PC-3 and PC-3M-IE8 detected by CCK8. **F** Histogram analysis of cell cycle analysis using PC-3M-2B4 and 22RV1 cells treated as indicated. **G** The representative peptide of p16 from mass spectrometry of p16 IP in two PCa cells. *$p < 0.05$ and **$p < 0.01$

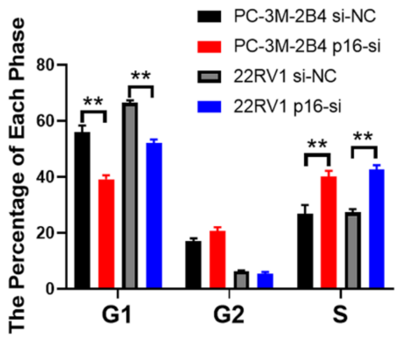

Supplemental Fig. 5 The histogram analysis of p16 on cell cycle in PCa cells

Histogram analysis of cell cycle analysis using PC-3M-2B4 and 22RV1 cells treated as indicated. *$p < 0.05$ and **$p < 0.01$

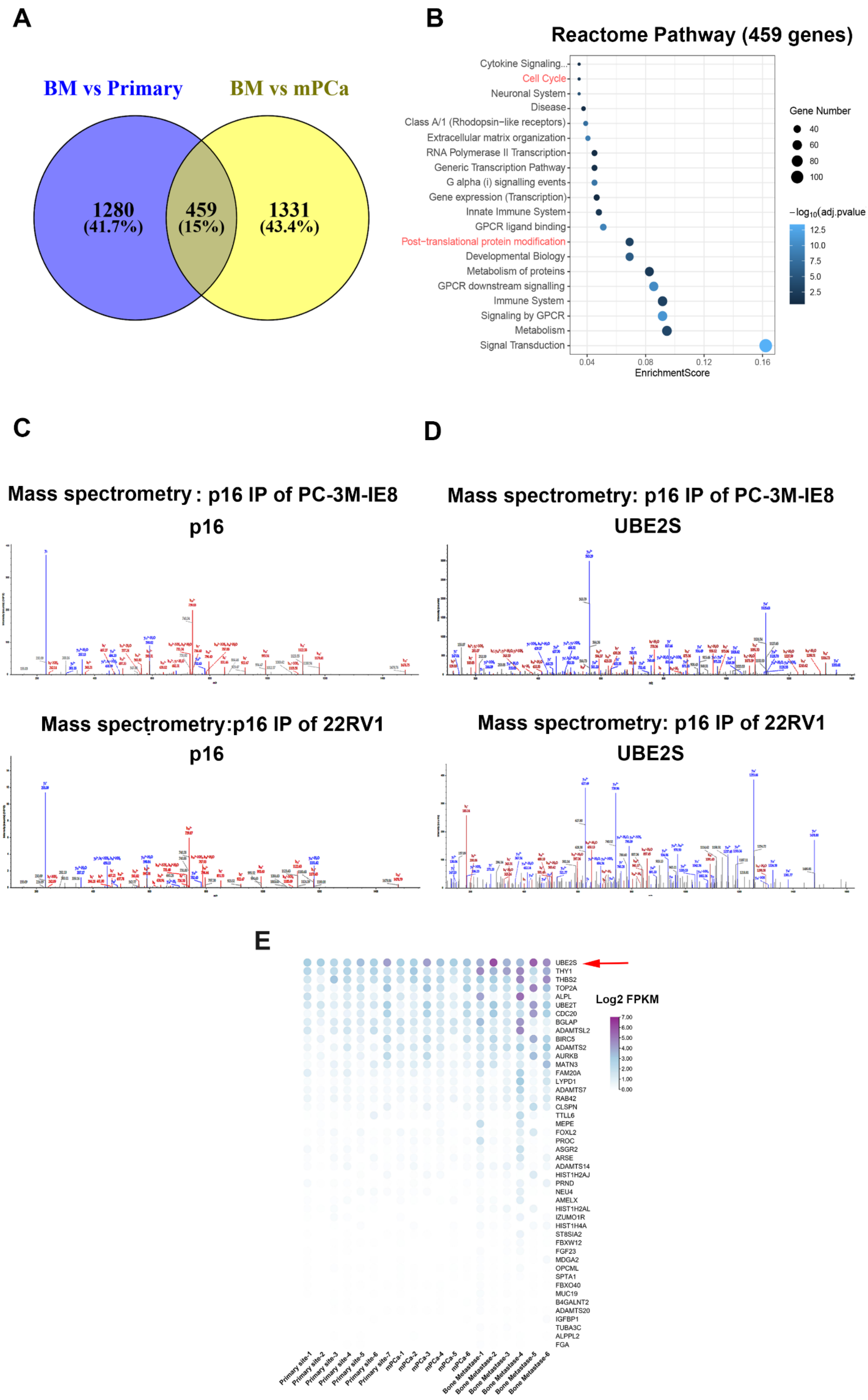

A
BM vs Primary
BM vs mPCa
1280
(41.7%)
459
(15%)
1331
(43.4%)
B
Reactome Pathway (459 genes)
Cytokine Signaling...
Cell Cycle
Neuronal System
Disease
Class A/1 (Rhodopsin−like receptors)
Extracellular matrix organization
RNA Polymerase II Transcription
Generic Transcription Pathway
G alpha (i) signalling events
Gene expression (Transcription)
Innate Immune System
GPCR ligand binding
Post−translational protein modification
Developmental Biology
Metabolism of proteins
GPCR downstream signalling
Immune System
Signaling by GPCR
Metabolism
Signal Transduction
0.04
0.08
0.12
0.16
EnrichmentScore
Gene Number
40
60
80
100
−log10(adj.pvalue)
12.5
10.0
7.5
5.0
2.5
C
Mass spectrometry : p16 IP of PC-3M-IE8
p16
Mass spectrometry:p16 IP of 22RV1
p16
D
Mass spectrometry: p16 IP of PC-3M-IE8
UBE2S
Mass spectrometry: p16 IP of 22RV1
UBE2S
E
UBE2S
THY1
THBS2
TOP2A
ALPL
UBE2T
CDC20
BGLAP
ADAMTSL2
BIRC5
ADAMTS2
AURKB
MATN3
FAM20A
LYPD1
ADAMTS7
RAB42
CLSPN
TTLL6
MEPE
FOXL2
PROC
ASGR2
ARSE
ADAMTS14
HIST1H2AJ
PRND
NEU4
AMELX
HIST1H2AL
IZUMO1R
HIST1H4A
ST8SIA2
FBXW12
FGF23
MDGA2
OPCML
SPTA1
FBXO40
MUC19
B4GALNT2
ADAMTS20
IGFBP1
TUBA3C
ALPPL2
FGA
Log2 FPKM
7.00
6.00
5.00
4.00
3.00
2.00
1.00
0.00
Primary site-1
Primary site-2
Primary site-3
Primary site-4
Primary site-5
Primary site-6
Primary site-7
mPCa-1
mPCa-2
mPCa-3
mPCa-4
mPCa-5
mPCa-6
Bone Metastase-1
Bone Metastase-2
Bone Metastase-3
Bone Metastase-4
Bone Metastase-5
Bone Metastase-6

Supplemental Fig. 6 UBE2S is identified to bind with p16 in PCa cells.

A Venn image showed that 459 genes were up-regulated in both BM vs Primary site group and BM vs mPCa group. **B** Bubble chart of Reactome pathway analysis of upregulated 459 genes in a. **C** The representative peptide of p16 from mass spectrometry of p16 IP in G1/S boundary synchronized PCa cells. **D** The representative peptide of UBE2S from mass spectrometry of p16 IP in G1/S boundary synchronized PCa cells. **E** Heatmap of post translation pathway enrich genes from b in each group.

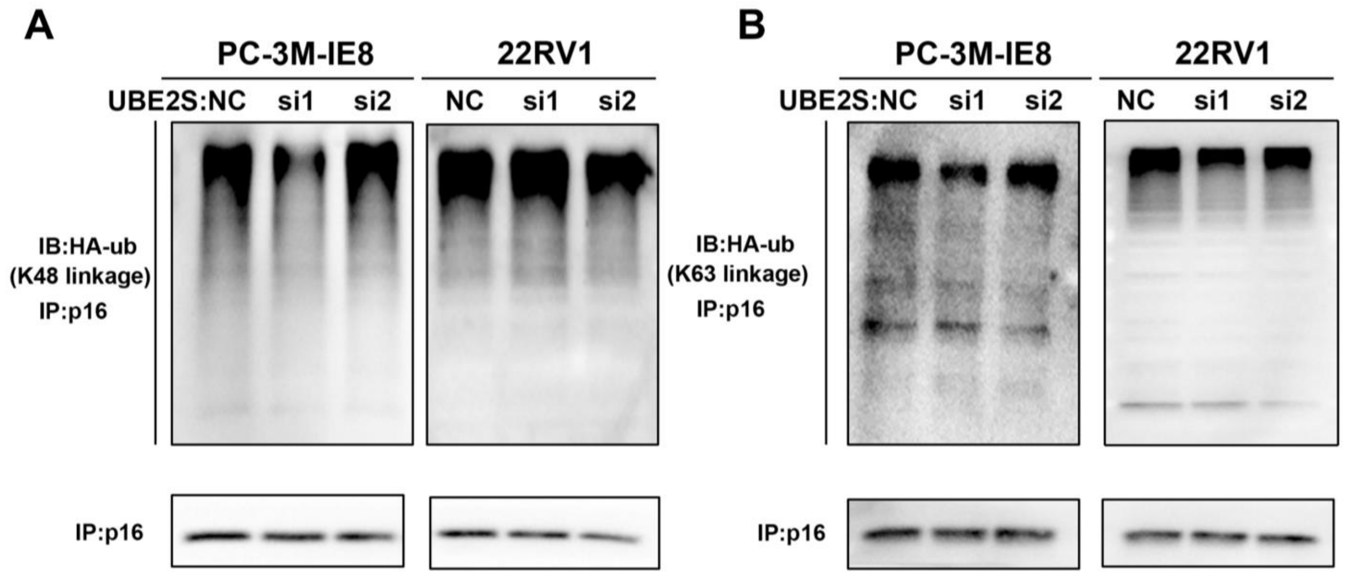

Supplemental Fig. 7 The K48 and K63-linkage ubiquitination of p16 in UBE2S-knockdown PCa cells.

A-B Co-IP analysis of ubiquitination of p16 in UBE2S-knockdown PC-3M-IE8 and 22RV1 transfected with K48 and K63 linkage HA-ub plasmid and synchronized in G1/S boundary, respectively.

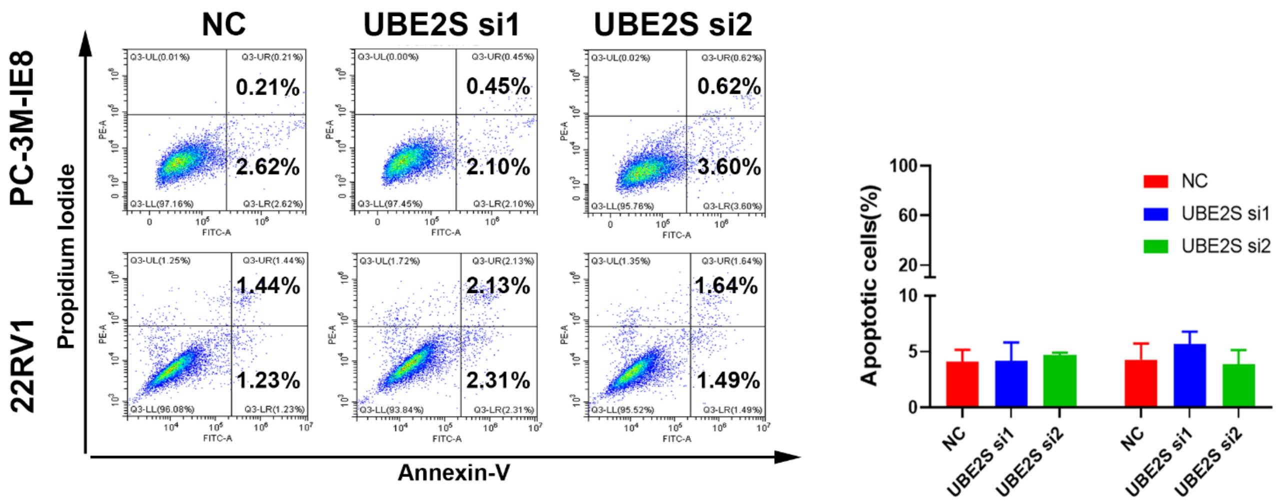

Supplemental Fig. 8 UBE2S has no effect on cell apoptosis in PCa cells.

Representative images and histogram analysis of cell apoptosis by flow cytometry using PC-3M-2B4 and 22RV1 cells treated as indicated.

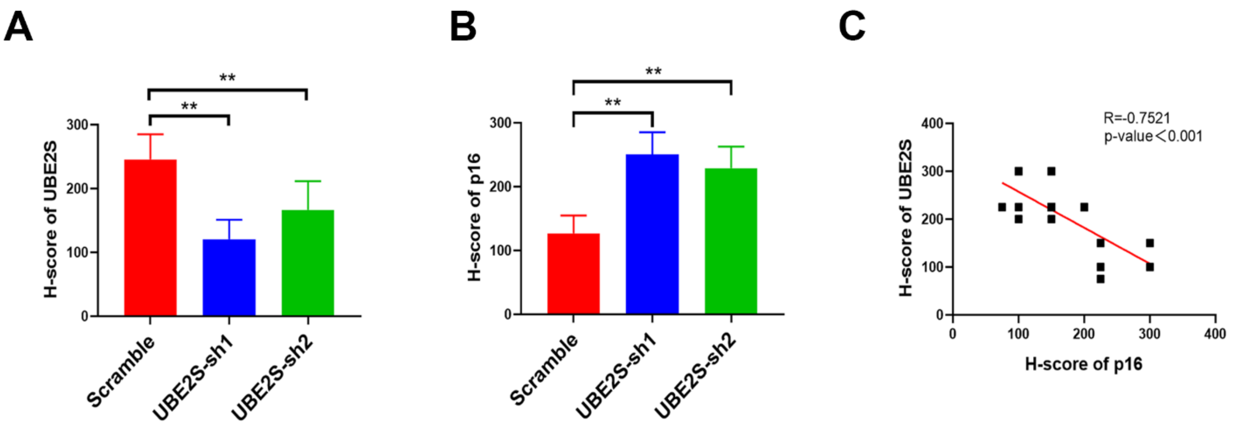

Supplemental Fig. 9 The correlation of UBE2S and p16 in nude mice.

A Histogram analysis of UBE2S staining in each group. **B** Histogram analysis of p16 staining in each group. **C** The correlation of UBE2S and p16 expression in the tumor from nude mice. *$p < 0.05$ and **$p < 0.01$

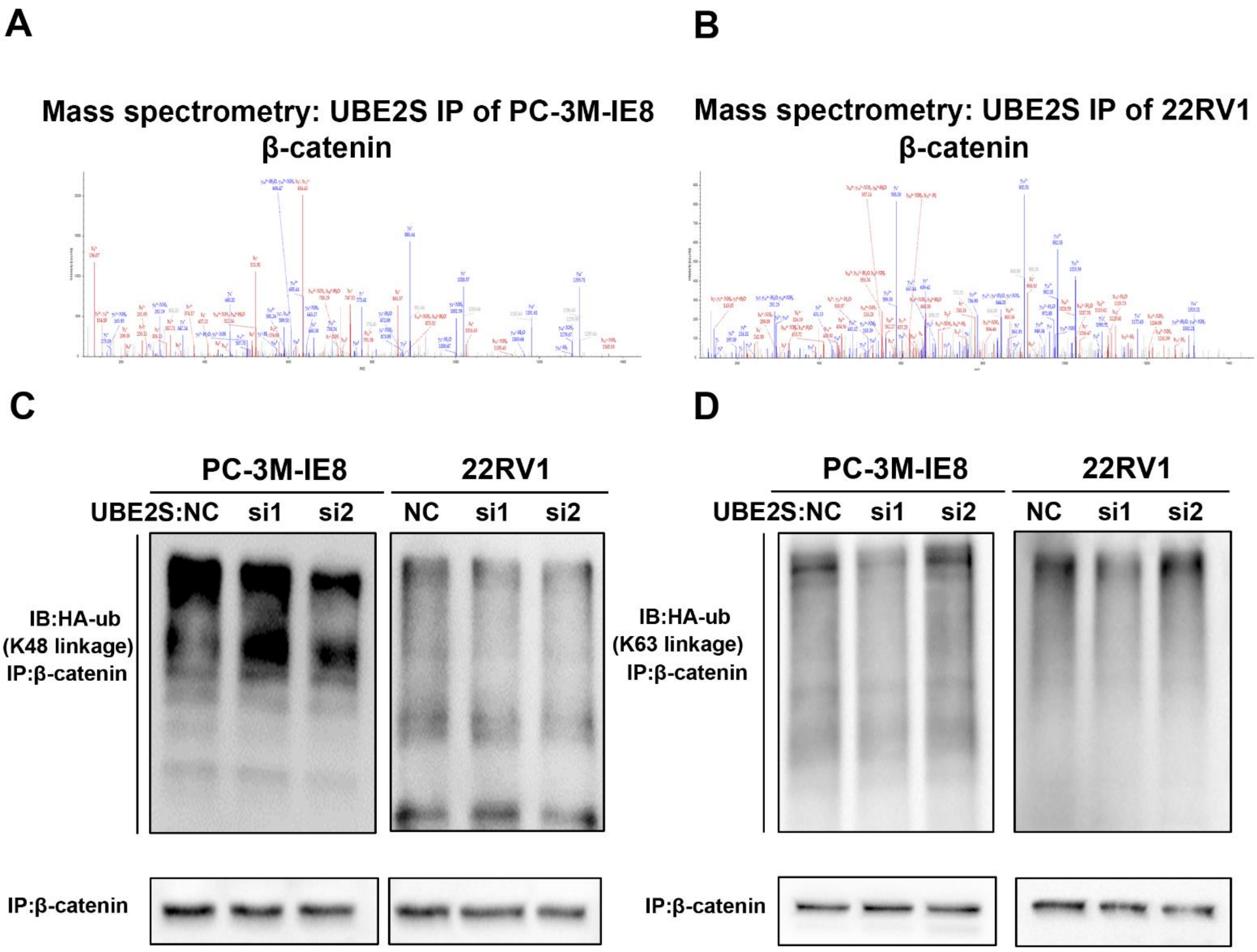

Supplemental Fig. 10 The K48 and K63-linkage ubiquitination of β-catenin in UBE2S-knockdown PCa cells.

A-B The representative peptide of β-catenin from mass spectrometry of UBE2S IP in PC-3M-IE8 (A) and

22RV1 (B). **C-D** Co-IP analysis of ubiquitination of β-catenin in UBE2S-knockdown PC-3M-IE8 and 22RV1 transfected with K48 and K63 linkage HA-ub plasmid, respectively.

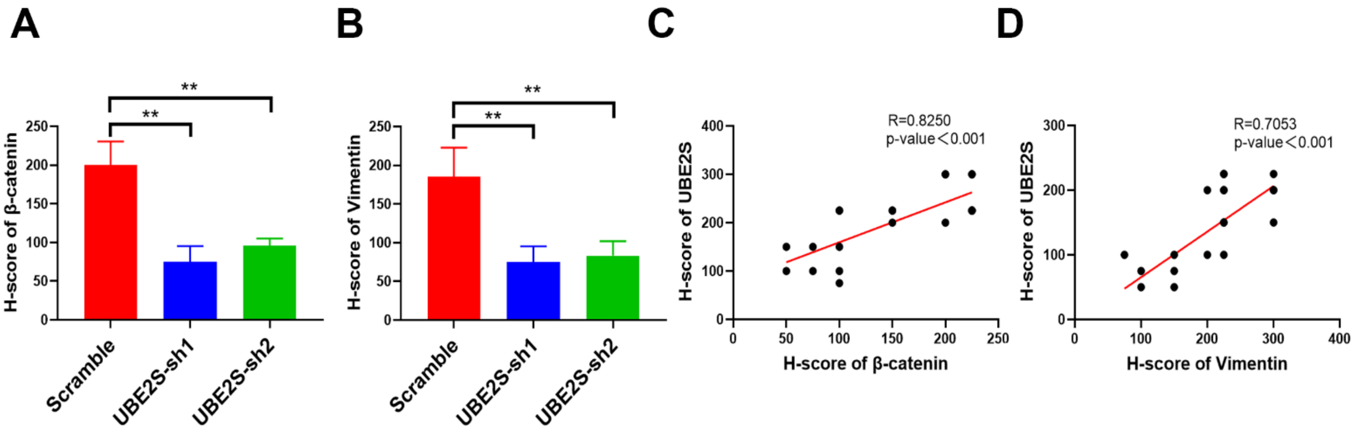

Supplemental Fig. 11 The correlation of UBE2S, β-catenin and Vimentin in nude mice.

A-B Histogram analysis of β-catenin and vimentin staining in each group. **C** The correlation of UBE2S and β-catenin expression in the tumor from nude mice. **D** The correlation of UBE2S and Vimentin expression in the tumor from nude mice. **p* < 0.05 and ***p* < 0.01

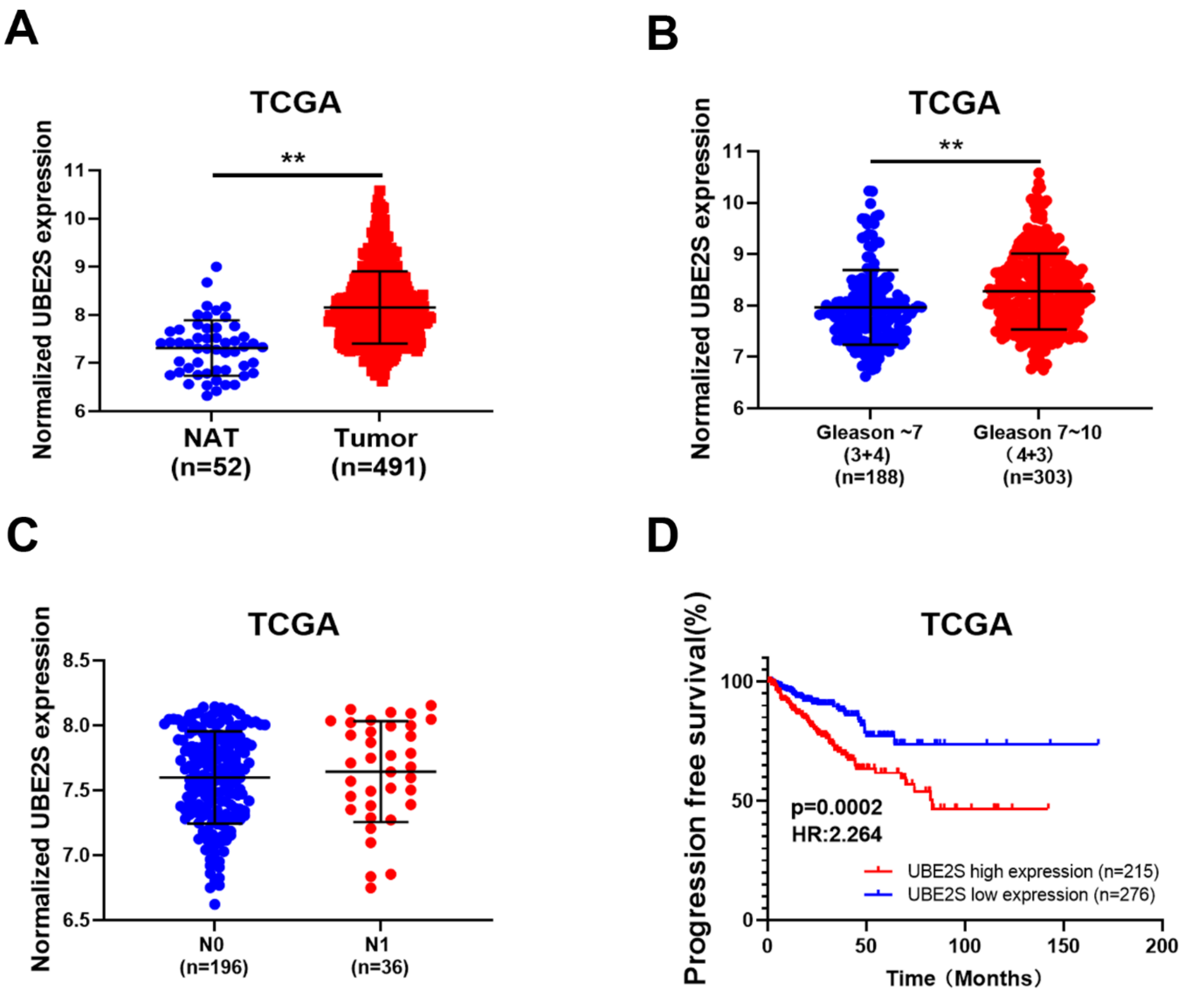

Supplemental Fig. 12 The expression of UBE2S is associated with advanced nodal metastasis, higher Gleason score and poor DFS in TCGA

A The expression difference of UBE2S between NAT and tumor tissues of PCa in TCGA PRAD. **B** The expression difference of UBE2S between low Gleason score (6-7(3+4)) PCa tissues and high Gleason score (7(4+3)-10) PCa tissues in TCGA PRAD. **C** The expression difference of UBE2S between lymph node-negative and lymph node-positive tissues of PCa in TCGA PRAD. **D** Kaplan-Meier curves for disease-free survival of PCa patients with high or low expression of UBE2S in TCGA PRAD. **p* < 0.05 and ***p* < 0.01

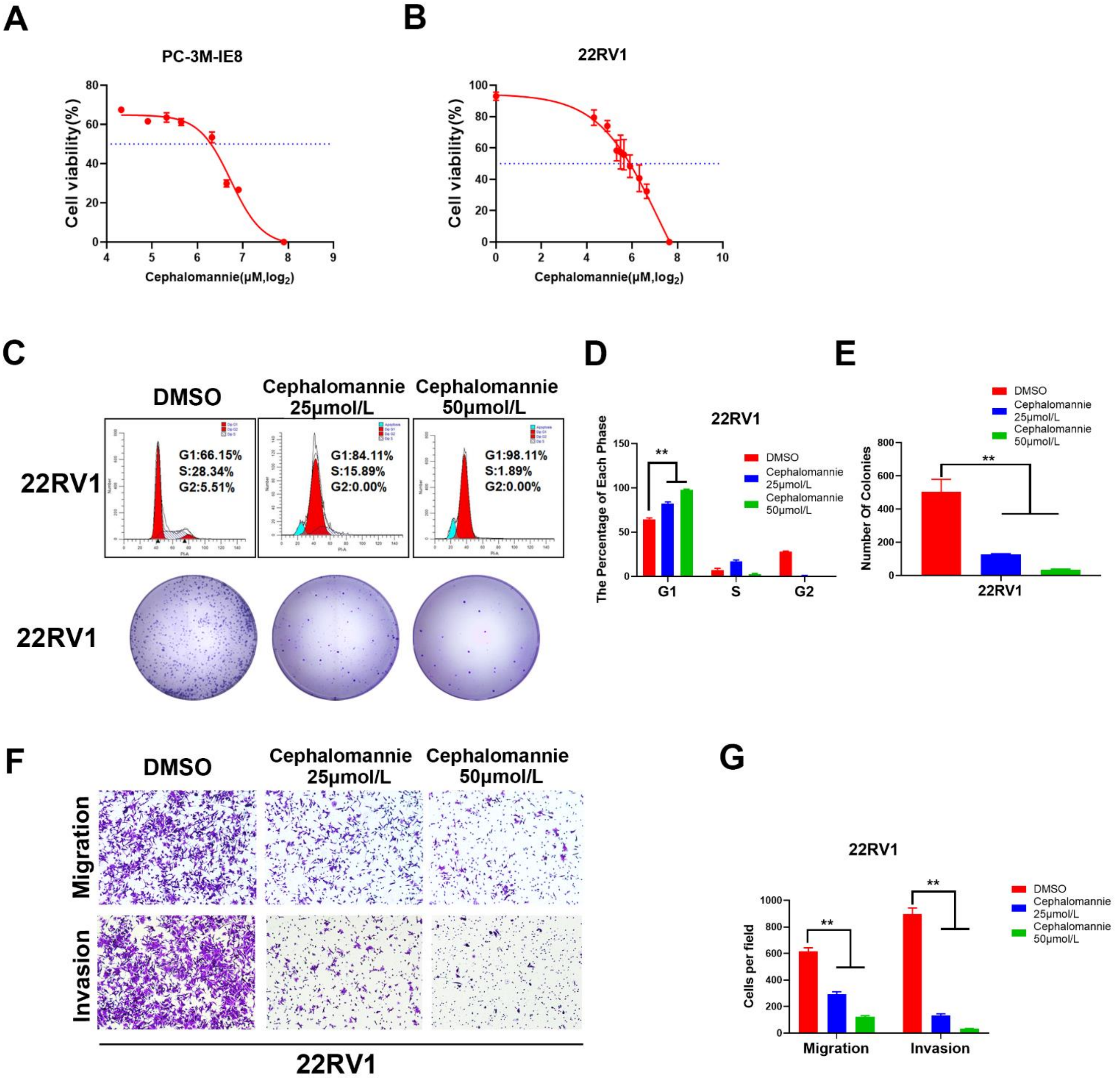

Supplemental Fig. 13 The effect of cephalomannie on PCa cells *in vitro.*

A-B Dose-reponse curves of PC-3M-IE8 (A) and 22RV1 (B) PCa cells treated with increasing concentrations of cephalomannie for 48 h. **C** Representative images of cell cycle analysis and colony formation assay of 22RV1 treated with varying concentrations of cephalomannine. **D-E** Histogram analysis of cell cycle analysis

(D) and colony formation (E) of 22RV1 treated with varying concentrations of cephalomannine. **F-G** Representative images (F) and histogram analysis (G) of migration and invasion assays of PC-3M-IE8 treated with varying concentrations of cephalomannine. *$p < 0.05$ and **$p < 0.01$

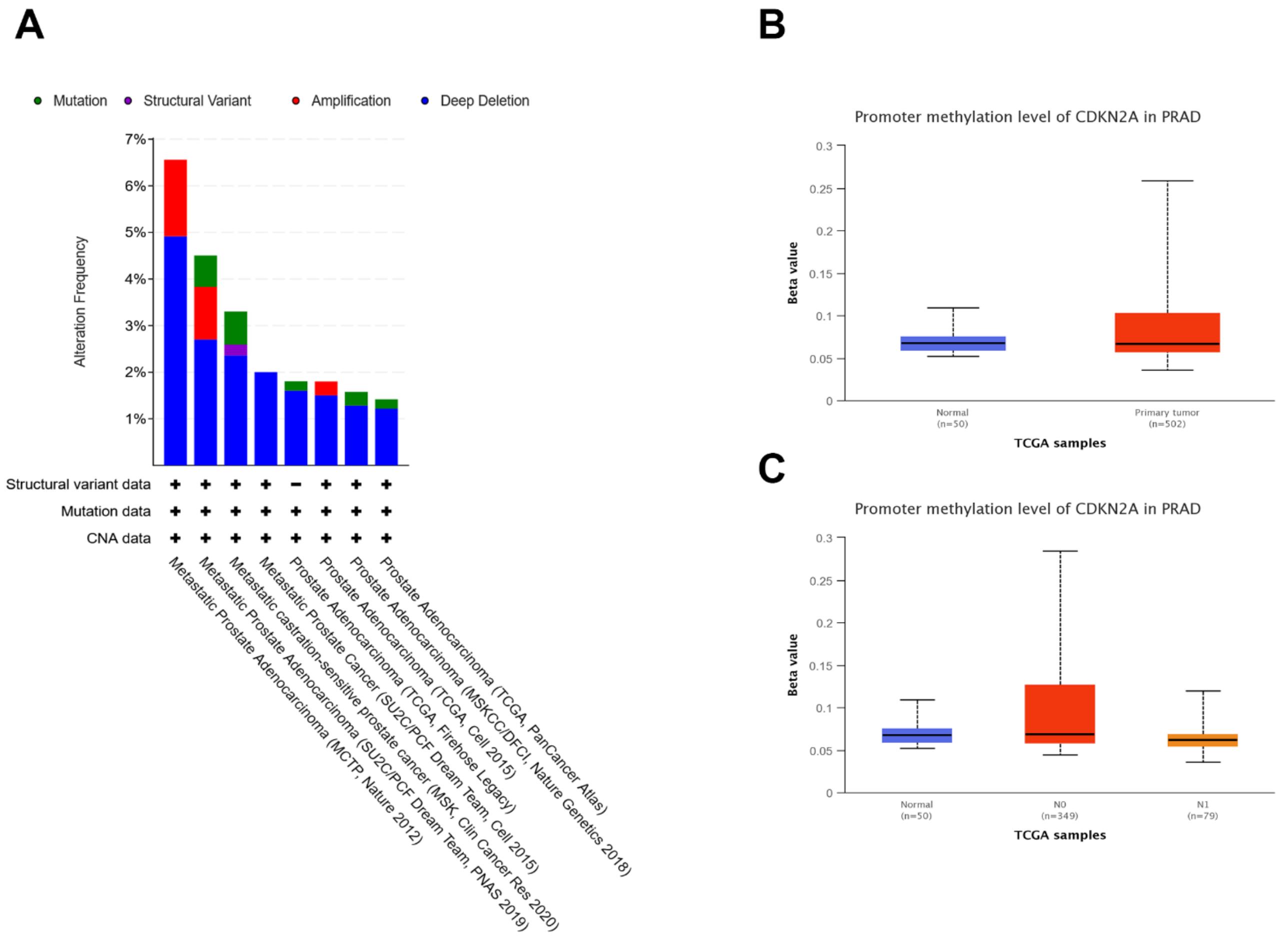

Supplemental Fig. 14 The genetic and epigenetic alterations of CDKN2A in public databases.

A The frequency of mutation, deep deletion, amplification and structural variant of CDKN2A in PCa and metastatic PCa in seven public databases. **B** The promoter methylation level of CDKN2A in TCGA-PRAD between NAT and Primary tumour. **C** The promoter methylation level of CDKN2A in TCGA-PRAD between NAT and different N-stage PCa.
